# Mutation of amphioxus *Pdx* and *Cdx* demonstrates conserved roles for ParaHox genes in gut, anus and tail patterning

**DOI:** 10.1186/s12915-020-00796-2

**Published:** 2020-06-16

**Authors:** Yanhong Zhong, Carlos Herrera-Úbeda, Jordi Garcia-Fernàndez, Guang Li, Peter W. H. Holland

**Affiliations:** 1 State Key Laboratory of Cellular Stress Biology, School of Life Sciences, Xiamen University, Xiamen, China; 2 Department of Zoology, University of Oxford, Oxford, OX1 3SZ UK; 3 Department of Genetics, Microbiology & Statistics, and Institute of Biomedicine (IBUB), University of Barcelona, 08028 Barcelona, Spain

**Keywords:** Cephalochordate, Branchiostoma, Homeobox, Cambrian, Endoderm, Tail, Retinoic acid, Hox cluster, Cyp26

## Abstract

**Background:**

The homeobox genes *Pdx* and *Cdx* are widespread across the animal kingdom and part of the small ParaHox gene cluster. Gene expression patterns suggest ancient roles for *Pdx* and *Cdx* in patterning the through-gut of bilaterian animals although functional data are available for few lineages. To examine evolutionary conservation of *Pdx* and *Cdx* gene functions, we focus on amphioxus, small marine animals that occupy a pivotal position in chordate evolution and in which ParaHox gene clustering was first reported.

**Results:**

Using transcription activator-like effector nucleases (TALENs), we engineer frameshift mutations in the *Pdx* and *Cdx* genes of the amphioxus *Branchiostoma floridae* and establish mutant lines. Homozygous *Pdx* mutants have a defect in amphioxus endoderm, manifest as loss of a midgut region expressing endogenous GFP. The anus fails to open in homozygous *Cdx* mutants, which also have defects in posterior body extension and epidermal tail fin development. Treatment with an inverse agonist of retinoic acid (RA) signalling partially rescues the axial and tail fin phenotypes indicating they are caused by increased RA signalling. Gene expression analyses and luciferase assays suggest that posterior RA levels are kept low in wild type animals by a likely direct transcriptional regulation of a *Cyp26* gene by Cdx. Transcriptome analysis reveals extensive gene expression changes in mutants, with a disproportionate effect of *Pdx* and *Cdx* on gut-enriched genes and a colinear-like effect of *Cdx* on Hox genes.

**Conclusions:**

These data reveal that amphioxus *Pdx* and *Cdx* have roles in specifying middle and posterior cell fates in the endoderm of the gut, roles that likely date to the origin of Bilateria. This conclusion is consistent with these two ParaHox genes playing a role in the origin of the bilaterian through-gut with a distinct anus, morphological innovations that contributed to ecological change in the Cambrian. In addition, we find that amphioxus *Cdx* promotes body axis extension through a molecular mechanism conserved with vertebrates. The axial extension role for *Cdx* dates back at least to the origin of Chordata and may have facilitated the evolution of the post-anal tail and active locomotion in chordates.

## Background

The discovery that three homeobox genes, *Gsx*, *Pdx* and *Cdx*, are organised in a small gene cluster raised the possibility that these genes might act in a coordinated way. This ParaHox cluster was first described in the amphioxus *Branchiostoma floridae* [1], and later in human, several other vertebrates, an echinoderm, a hemichordate and two molluscs [2–8]. The cluster has broken in many taxa through genomic rearrangements or gene losses [1, 3, 9, 10]. The *Pdx* gene, also called *Pdx1*, *Xlox*, *Lox*, *Ipf1*, *Idx1* or *Stf1*, is expressed in developing gut endoderm in vertebrates, with sharp anterior and posterior limits, and later in the pancreas and where the duodenum meets the stomach [11–13]. In amphioxus embryos, *Pdx* is also expressed strongly in the midgut endoderm and, puzzlingly, in two putative receptor cells in the neural tube [1, 14]. The latter lie adjacent to a large pigment cell [1, 15] and comprise the first photosensory organ of Hesse (dorsal ocellus) to develop [16]. The amphioxus *Cdx* gene is also expressed in the gut, more posteriorly where the anus will form, and in other posterior tissues at early developmental stages [1]. Similarly, vertebrate *Cdx* genes are expressed caudally, including the posterior gut, although multiple paralogues have subtly different patterns and additional expression sites [17–19]. *Gsx* is expressed primarily in neural tissue in amphioxus, *Drosophila* and mouse [1].

Together with data from echinoderms, annelids and molluscs, these patterns led to the hypothesis that ParaHox genes were components of an ancient system for patterning the bilaterian gut [1, 20–23]. The origin of a ‘through-gut’, with the mouth, digestive regions and anus, may have facilitated the evolution of predation and active burrowing, key drivers of animal diversification in the Cambrian [1, 20, 24] (but see [25]). To test this hypothesis, we need to know if ParaHox genes consistently specify region-specific fate, instructing cells to differentiate according to their head to tail position. Such a role has been suggested for vertebrate *Pdx*. Mutation of both *Pdx* alleles results in the absence of a pancreas in mice and humans [12, 26–28], conditional deletion in mouse causes homeotic-like transformation of endoderm cells [13], and ectopic expression in chick causes endoderm cells to change molecular identity and behaviour [29]. *Pdx* has a similar role in sea urchin larvae, where morpholino knockdown inhibits the formation of the sphincter between the midgut and hindgut [22, 30]. In adult mammals, the Pdx protein is a transcriptional activator of *insulin* and other genes in β cells of the endocrine pancreas [31]. No insights are possible from *Drosophila* or nematodes as they have lost *Pdx*, while functional data are difficult to obtain in other invertebrates.

For *Cdx* genes, there is evidence for a role in gut patterning in several taxa. For example, heterozygous mutation of mouse *Cdx2* causes homeotic-like transformation of posterior gut cells [32] and inhibiting *Cdx* function in sea urchin development allows posterior gut cells to express a more anterior marker [22]. In *Drosophila*, *Cdx* is necessary for the invagination of the hindgut [33], and *Caenorhabditis elegans Cdx* has roles in the development of the rectum [34]. A second role for *Cdx* genes in body axis or tail extension is also seen in some taxa, including mice, *Xenopus* and zebrafish [18, 35–37], an ascidian [38], and short-germ arthropods *Artemia*, *Tribolium* and *Gryllus* [39, 40]. There is no similar function in *Drosophila*, however, which may be because *Cdx* was recruited into a novel gene network patterning the rapidly developing long-germ band embryo [41]. While roles in the gut and tail may be shared between distant phyla, there are also taxon-specific roles. Thus, mutations in mouse *Cdx1* or *Cdx2* cause homeotic transformations of mesodermally derived vertebrae, at least in part by altering Hox gene expression [18, 42], and interfering with *Cdx* function in *Xenopus* causes disruption to neural patterning [37, 43]. In *Drosophila*, *Cdx* also specifies fate of the cells forming the anal plates on the exterior of the most posterior segment [44]. Similarly, many cell types are affected by mutation of *Cdx* (*pal-1*) in the nematode *Caenorhabditis elegans*, including posterior epidermal rays [45].

Expression of *Gsx* is variable between taxa, making inference of its ancestral role in bilaterians difficult. The larvae of some annelids and a mollusc show clear *Gsx* expression around the forming mouth, consistent with a general link between ParaHox genes and the through-gut [9, 23, 46]. This pattern, however, is not seen in all molluscs nor in ecdysozoans [47]. Wollesen et al. [47] have therefore argued that *Gsx* expression in the anterior nervous system was more likely an ancestral bilaterian trait, with recruitment into foregut a derived character of some taxa. Either way, deuterostomes such as amphioxus are probably not suitable for inferring ancestral roles of *Gsx* as the deuterostome mouth may not be homologous to the mouth of ancestral bilaterians [20].

Experimental data from a diversity of invertebrates are necessary to evaluate which roles of *Pdx* and *Cdx* are conserved between taxa and to evaluate their ancestry. Key questions are whether these genes specify region-specific cell fate in the gut and formation of the anus. Since there is no living outgroup to bilaterians that possesses a homologous through-gut, it is necessary to address this question using ingroups from each bilaterian clade. The recent development of TALEN mutagenesis methods applicable to amphioxus has opened up an opportunity to investigate these functions in this animal occupying a pivotal phylogenetic position [48, 49].

## Results

### Generation of stable *Pdx* and *Cdx* mutant lines in amphioxus

**Fig. 1:**
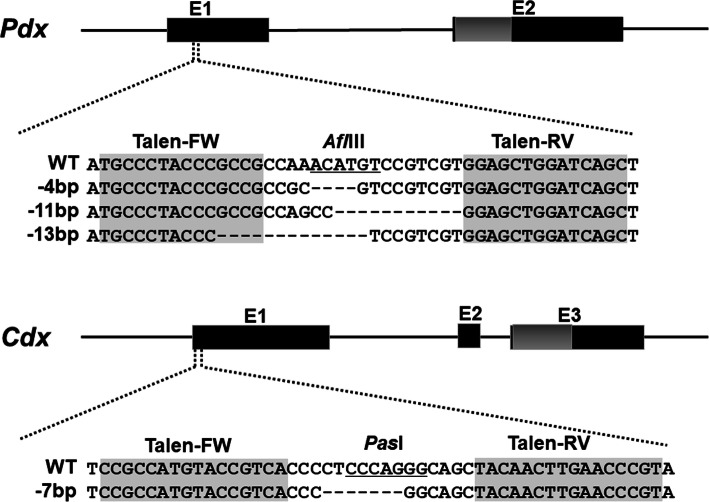
Gene structure, sequences targeted by TALENs and mutations generated. E1, E2, E3, coding regions of exons; grey, homeobox; WT, wild type sequence

We deduced gene structures for amphioxus *Pdx* and *Cdx* and designed constructs encoding TALEN pairs targeted to the first coding exon of each (Fig. 1; Additional file 1: Figs. S1-S6). For each gene, two TALEN mRNAs were co-injected into unfertilised eggs of *B. floridae* and successful mutagenesis detected by PCR on pools of neurula stage embryos (Additional file 1: Table S1, Fig. S7). Remaining embryos were reared to maturity to generate mosaic F0 animals; founders were spawned and mutations typed by PCR on sperm or pools of neurulae generated by outcrossing. Embryos from F0 × wild type crosses were reared to maturity to generate F1 heterozygous mutants; these lines were expanded by crossing with wild type animals, rearing embryos to maturity and intercrossing offspring.

We generated amphioxus lines with 4-bp, 11-bp and 13-bp deletions (4Δ, 11Δ, 13Δ) in *Pdx* (Fig. 1). Each deletion causes a frameshift predicted to give a protein comprising 31–33 amino acids of wild type protein followed by peptide sequence with no strong similarity to any known protein (Additional file 1: Fig. S8). We generated a *Cdx* mutant line with a 7-bp deletion (7Δ) predicted to give a short peptide comprising the first 5 amino acids of *Cdx* plus 4 additional amino acids before a stop codon (Fig. 1; Additional file 1: Fig. S9). None of the predicted products contains a homeodomain.

### *Pdx* is necessary for correct spatial differentiation of the midgut

**Fig. 2:**
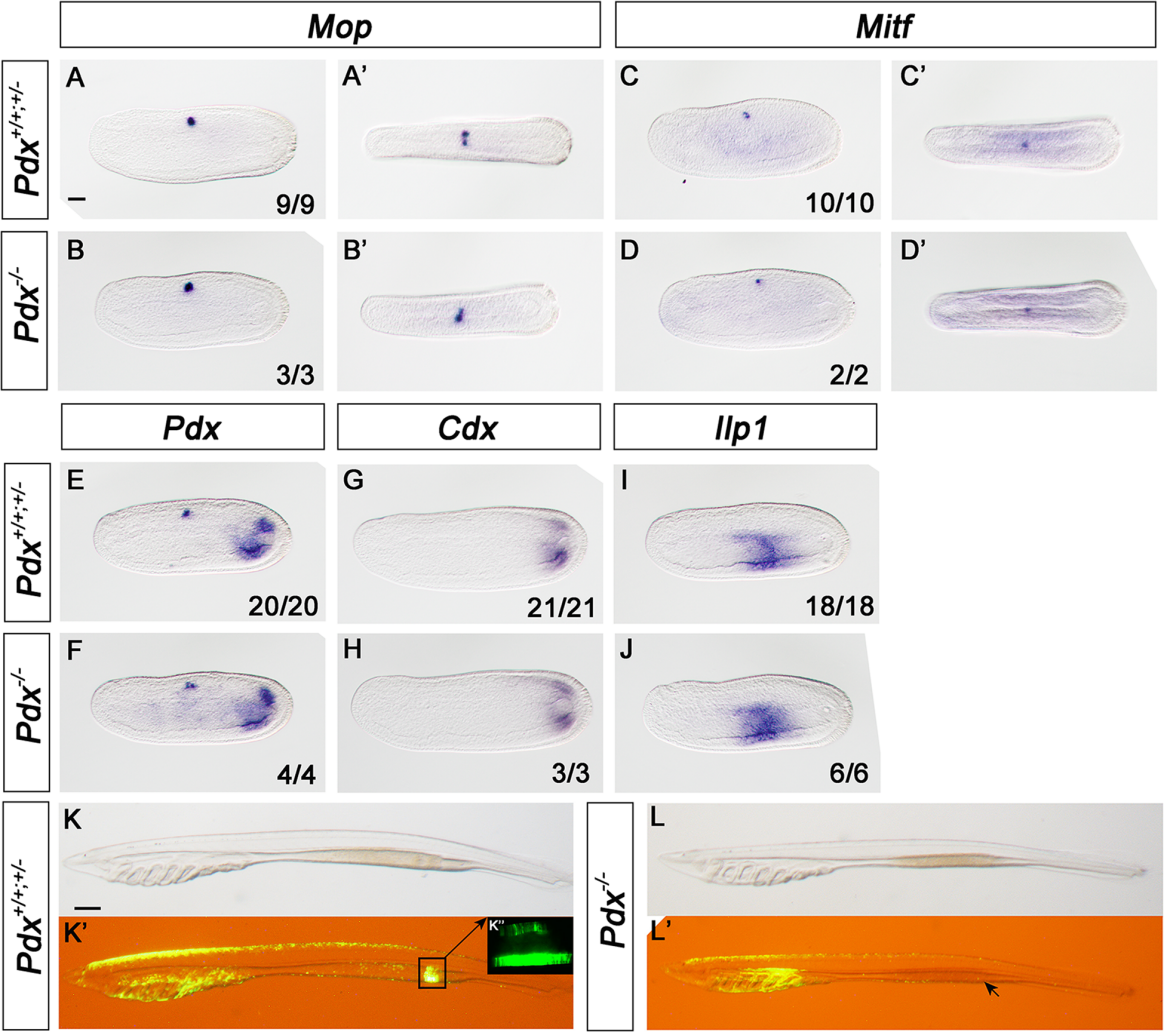
*Pdx* mutant phenotype. In situ hybridisation to wild type and *Pdx* 4Δ homozygous mutant neurulae stage (13 h) amphioxus embryos for *Mop* (**a**, **b**), *Mitf* (**c**, **d**), *Pdx* (**e**, **f**), *Cdx* (**g**, **h**) and *Ilp1* (**i**, **j**). Embryos were genotyped by PCR after hybridisation. Green fluorescence in 7 gill slit amphioxus larvae showing endodermal patch in wild type (**k**, **k’**) but not homozygous *Pdx* 4Δ mutants (**l**, **l’**). Inset **k”** shows confocal imaging of sibling larva showing fluorescence in endoderm cells. Anterior to the left in all images; dorsal to the top in all cases except **a’**, **b’**, **c’** and **d’** which are dorsal views with the right side to the top of the image. Scale bar in **a**, 50 μm, refers to **a** to **j**; scale bar in **k**, 200 μm, refers to **k** to **l’**

Embryos derived from crosses between heterozygous *Pdx* 4Δ mutants, and crosses between *Pdx* mutant lines, generated pools of morphologically identical embryos; genotyping revealed these include homozygotes, heterozygotes and wild type with no significant reduction in frequency of homozygous mutants (18/96; chi square *p* = 0.16). Since *Pdx* is strongly expressed in the gut and the first Hesse organ receptor cells in 13-h neurulae, we tested whether marker genes for these tissues were affected in *Pdx* mutants. In situ hybridisation revealed no difference in expression of *Mop* (*melanopsin*, expressed in the Hesse organ receptors; Fig. 2a, b) or *Mitf* (expressed in the adjacent pigment cell; Fig. 2c, d). Dorsal views show that the two receptors cells (Fig. 2a’, b’) and single pigment cell (Fig. 2c’, d’) are present in mutants. We also detected no difference in expression pattern in *Pdx* itself, *Cdx* or *Ilp1* (insulin-like peptide gene) in the endoderm of neurulae (Fig. 2e–j). Later in development, however, a morphological phenotype was visible in mutants when viewed under fluorescent illumination. In wild type and heterozygote larvae, endogenous green fluorescence is detected in cirri around the mouth and in a clearly delineated patch of the midgut endoderm, noted here for the first time. In contrast, in 4Δ/4Δ, 4Δ/11Δ and 11Δ/13Δ mutant larvae, clear fluorescence is seen only in buccal cirri. The difference is first detectable at the 5 gill slit stage (6 to 16 days development depending on batch) and persists to larvae with many gill slits (around 46 days; Fig. 2k, l, Additional file 1: Fig. S10, S11).

### *Cdx* is necessary for the formation of the anus and posterior axial extension

**Fig. 3:**
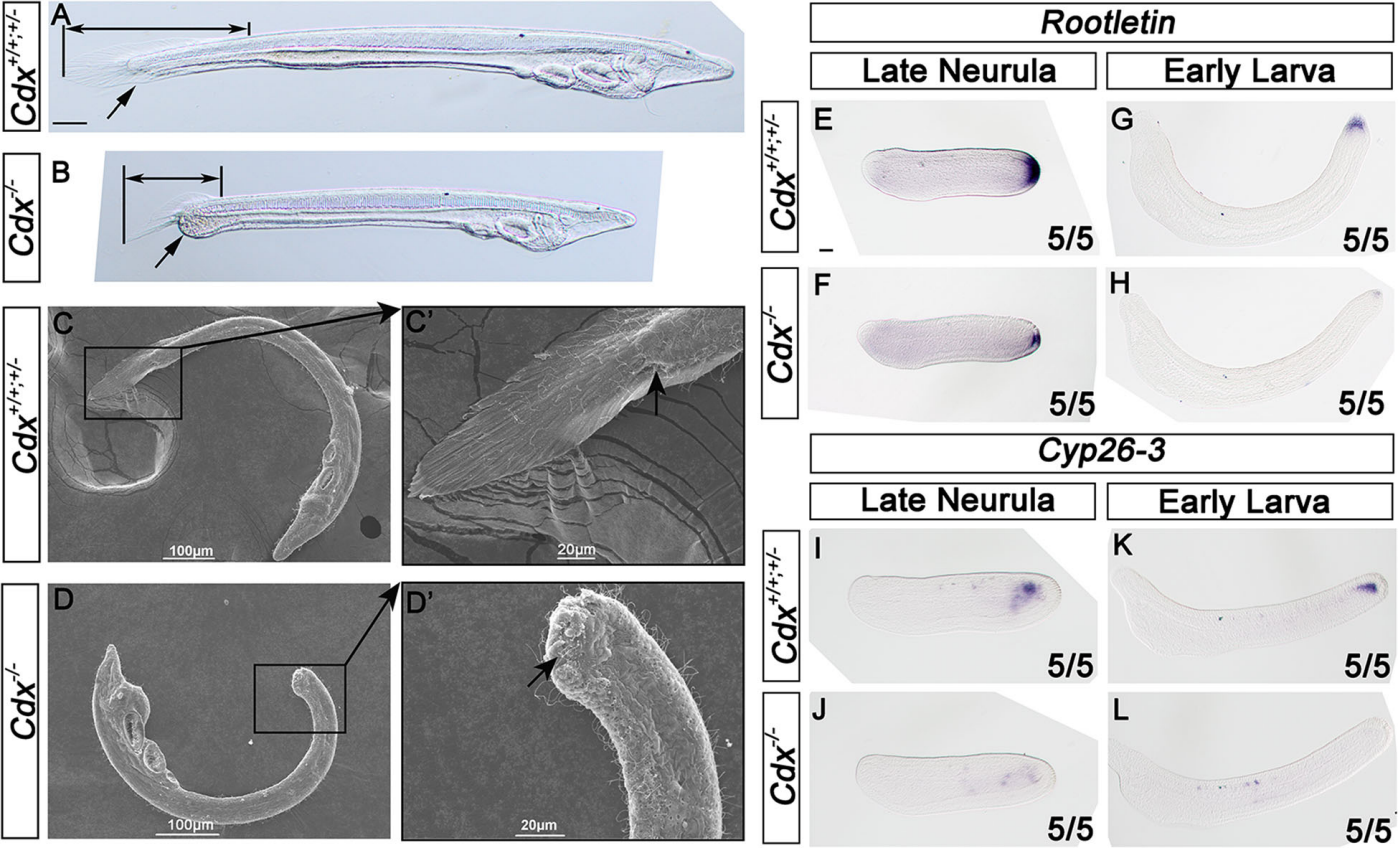
*Cdx* mutant phenotype. **a** Wild type and **b** homozygous mutant 3 gill slit (40 h) larvae showing the anus or lack of the anus (arrow) and distance from gut restriction to the tip of the tail (double-headed arrow). Scanning electron micrographs showing the anus and tail fin in 40-h wild type larva (**c**, **c’**) and closed anus and reduced tail fin in mutant larva (**d**, **d’**). In situ hybridisation to *Rootletin* RNA in **e** late neurula (16 h) wild type embryo, **f** late neurula (16 h) homozygous mutant embryo, **g** early larva (21 h) wild type embryo and **h** early larva (21 h) homozygous mutant embryo. In situ hybridisation to *Cyp26-3* RNA in **i** late neurula (16 h) wild type embryo, **j** late neurula (16 h) homozygous mutant embryo, **k** early larva (21 h) wild type embryo and **l** early larva (21 h) homozygous mutant embryo. Anterior to the right, dorsal to the top of the image in **a**–**c**. Anterior to the left, ventral to the top in **d**. Anterior to the left, dorsal to the top in **e** to **l**. Scale bar in **a**, 50 μm, refers to **a** and **b**; scale bar in **e**, 50 μm, refers to **e** to **l**.

Homozygous *Cdx*^−/−^ mutants are identical to wild type amphioxus in external appearance through gastrula and neurula stages, and until the mouth opens around 24 h post-fertilisation (Fig. 3; Additional file 1: Fig. S12). Starting at the 2 to 3 gill slit stage (30 to 40 h post-fertilisation), a morphological difference gradually begins to be evident as truncation of the body axis in homozygous mutants (Fig. 3a, b). As development proceeds, the posterior of the animal continues to extend only in wild type and heterozygous larvae, enhancing the phenotypic difference. By the 4 gill slit stage, homozygous mutants are approximately half the length of wild type larvae (Additional file 1: Fig. S13).

A second morphological difference becomes evident around the 3 gill slit stage in the gut. In homozygous mutants, the gut remains closed by the epithelial cell layer of the endoderm; in wild type animals, the anus perforates to generate the through-gut (Fig. 3c, d). When cultured algal cells are provided to 2-day and 3-day larvae, mutant larvae take up food material and in some animals the closed anus ruptures leaving a ragged terminus. The tail fin is also markedly reduced in mutants and develops from a smaller zone of the posterior ectoderm; this effect is most marked ventrally (Fig. 3a, b). The development of the amphioxus tail fin is driven by the extension of specialised epidermal cells containing long intracellular ciliary rootlets containing a coiled-coil protein Rootletin [50–52]. We find the *Rootletin* gene is expressed by fewer cells in mutant than in control sibling larvae (Fig. 3e–h). This alteration of *Rootletin* gene expression is consistent with a smaller number of epidermal cells being specified to differentiate into tail fin in *Cdx* mutant animals. The expression of a posteriorly expressed gene *Cyp26-3*, encoding a retinoic acid-degrading enzyme, is also greatly reduced in *Cdx* mutant embryos (Fig. 3i–l). We infer that *Cdx* is necessary for fate specification of posterior ectoderm cells, for formation of the anus and for tail extension.

### *Cdx* acts via the retinoic acid pathway in amphioxus posterior development

The imperforate anus, tail truncation, reduced *Rootletin* expression and tail fin phenotype observed in *Cdx* mutant larvae are similar to a suite of changes caused by exogenous treatment of amphioxus larvae with retinoic acid (RA) [51, 53]. We therefore asked whether *Cdx* mutation caused these phenotypes through an increase in RA in the posterior region.

**Fig. 4:**
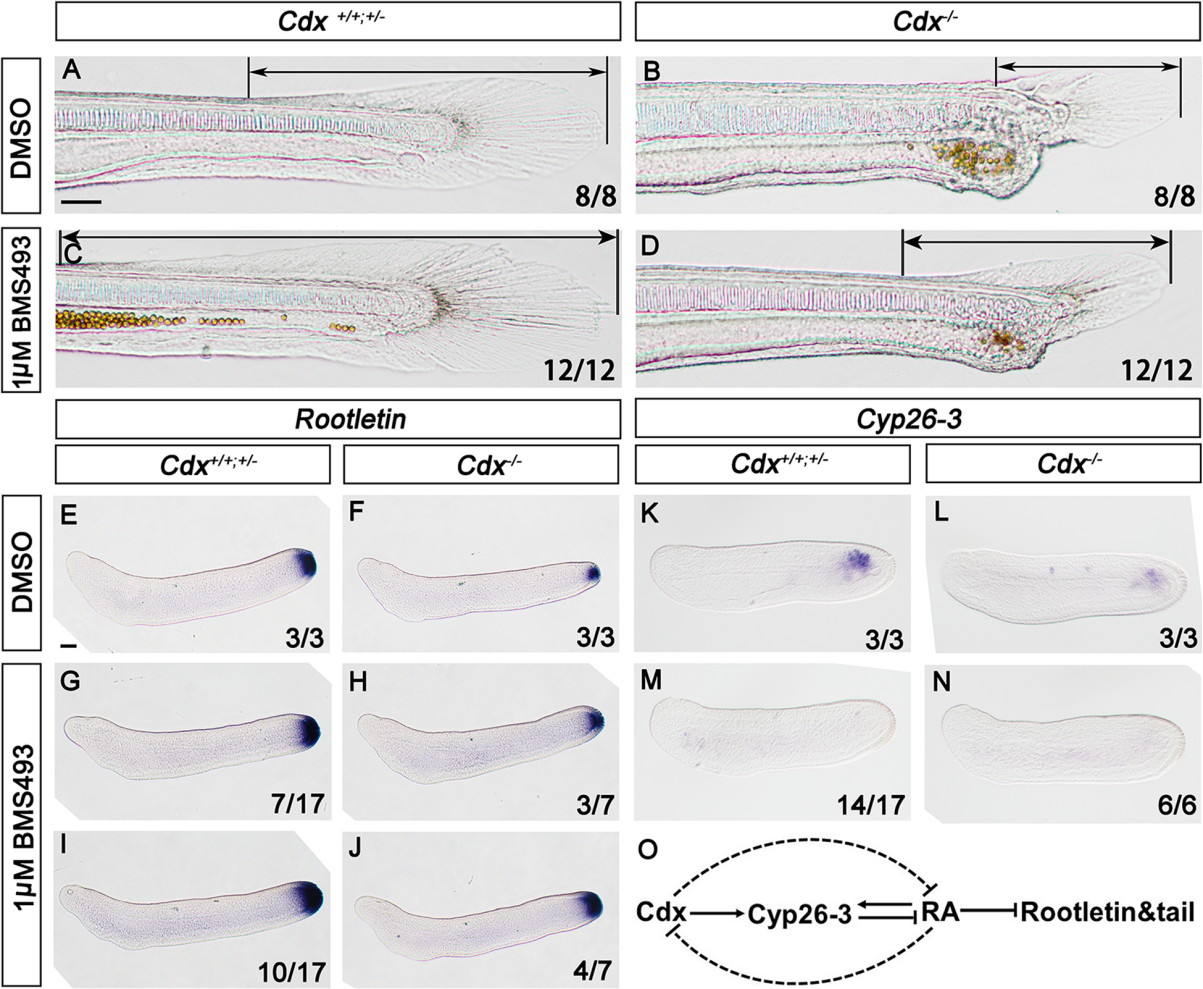
Inhibiting RA pathway partially rescues *Cdx* mutant phenotype. The large tail fin evident in wild type and *Cdx*^*+/−*^ heterozygous (‘normal’) amphioxus larvae (**a**, 30 h) is severely truncated in *Cdx*^*−/−*^ homozygous mutants and develops from fewer epidermal cells (**b**). Inhibition of the RA pathway using BMS493 enlarges the tail fin of normal larvae (**c**) and partially rescues the truncation in *Cdx*^*−/*−^ mutants (**d**). Tail fin development is presaged by the expression of the *Rootletin* gene in 18-h late neurula embryos, visualised by in situ hybridisation (**e**), which is also downregulated in *Cdx*^*−/−*^ mutants (**f**). Inhibition of the RA pathway results in the upregulation of *Rootletin* RNA to varying degrees in normal (**g**, **i**) and mutant (**h**, **j**) embryos. In contrast, posterior expression of *Cyp26-3* in 16-h embryos (**k**) is downregulated in *Cdx*^*−/−*^ mutants (**l**) and by inhibition of RA action (**m**, **n**). These data are consistent with a model (**o**) involving inhibition of RA signalling by *Cdx* and a feedback loop; interactions in dotted lines are either not confirmed (Cdx effect on RA independent of Cyp26-3) or deduced from previous work (RA inhibition of *Cdx* [14]). Anterior to the left, dorsal to the top in all images. Scale bar in **a**, 50 μm, refers to **a**–**d**; scale bar in **e**, 50 μm, refers to **e**–**n**

To test if dampening RA signalling partially rescues the mutant phenotype, we treated embryos derived from *Cdx* heterozygote crosses with BMS493, an inverse agonist of the pan-retinoic acid receptor (RAR; Fig. 4). Homozygous mutant larvae treated with BMS493 had enlarged tail fins compared to untreated mutants, especially dorsally, with the fin developing from more epidermal cells (Fig. 4b, d). Posterior growth of the body increased marginally but the anus did not open. In wild type and heterozygous embryos, BMS493 treatment gave the opposite phenotype to *Cdx* mutation (Fig. 4a, c): an enlarged larval tail fin derived from a larger region of posterior ectoderm (see also [53]). These phenotypes were presaged by subtle changes to expression of the *Rootletin* gene at earlier developmental stages (Fig. 4e–j). We infer, therefore, that the role of *Cdx* in tail fin formation and tail extension acts, at least in part, through the RA pathway.

**Fig. 5:**
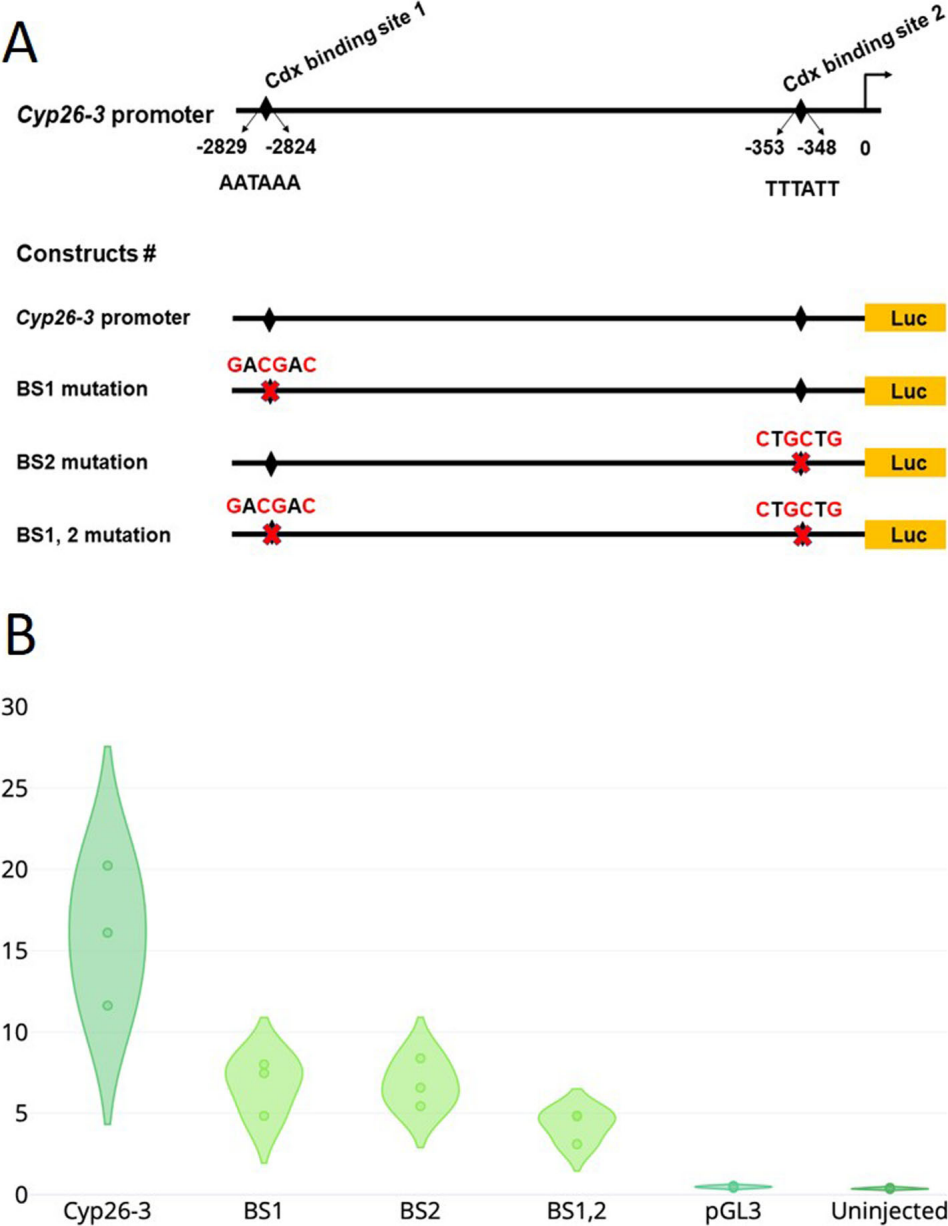
Reporter gene analysis of *Cyp26-3*. **a** Region 5′ of *Cyp26-3* gene showing two putative Cdx-binding sites (BS1, BS2). Numbers are distance before start codon. Four reporter gene constructs were tested in amphioxus embryos with 0, 1 or 2 Cdx sites mutated. **b** Violin plot showing relative levels of luciferase expression for each construct, a control luciferase vector pGL3 without *Cyp26-3* sequence, and uninjected control (values in Additional file 1: Table S4)

We examined a key player in the RA pathway, a cytochrome P450 family 26 (*Cyp26*) gene encoding an enzyme that degrades and clears excess RA [54]. Amphioxus has three *Cyp26* genes, derived from cephalochordate-specific duplication [53, 55, 56] (Additional file 1: Fig. S14, S15). In *B. floridae*, we find that *Cyp26-3* is expressed posteriorly at the neurula stage (Fig. 4k), unlike the pattern previously reported in *B. lanceolatum* [53]. In homozygote *Cdx* mutants, posterior *Cyp26-3* expression is downregulated but not abolished (Fig. 4l); treatment with BMS493 also downregulates *Cyp26-3* expression, to a more extreme degree (Fig. 4m, n). To investigate if *Cyp26* genes are direct transcriptional targets of Cdx in amphioxus (as in mouse [57]), we injected unfertilised amphioxus eggs with a luciferase reporter gene construct driven by 3.1 kb of *Cyp26-3* upstream sequence and assayed luciferase at the late neurula stage. The sequence includes several putative Cdx-binding sites (Additional file 1: Fig. S16) of which two have a close match to a consensus [58]; mutation of either or both decreases luciferase activity consistent with Cdx-binding positively regulating *Cyp26-3* expression (Fig. 5; Additional file 1: Tables S3, S4).

These results suggest that a major role of *Cdx* expression is to ensure RA activity is kept at a low level in the posterior part of the amphioxus embryo, at least in part via positive regulation of *Cyp26-3*. Downregulation of *Cyp26-3* expression by BMS493 is consistent with a feedback loop between RA and *Cyp26* activity as previously shown in zebrafish [59–61] (Fig. 4o).

### Effects of Cdx and Pdx mutation on amphioxus transcriptome

**Fig. 6:**
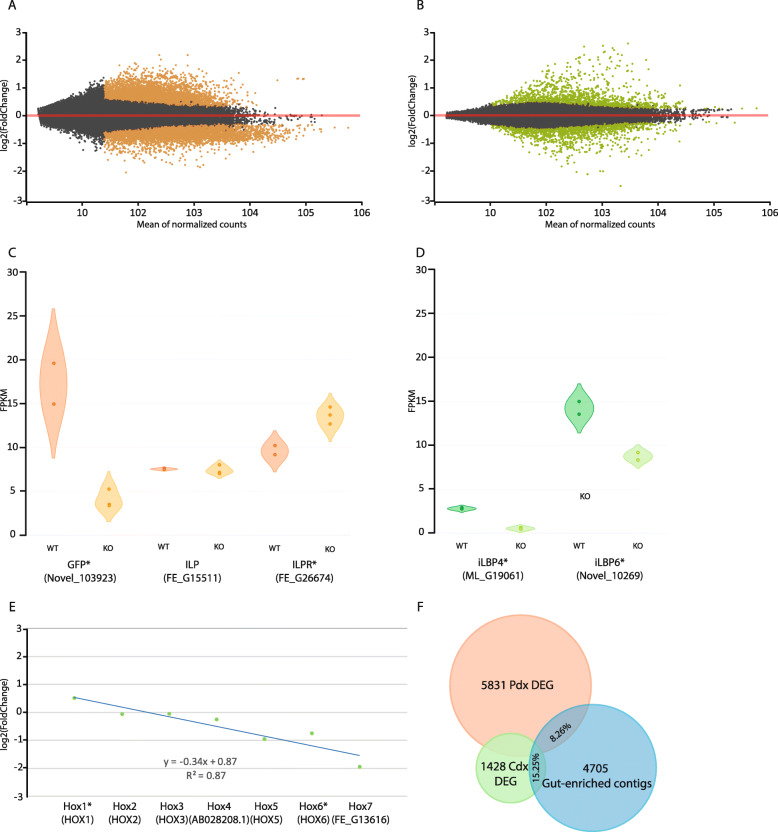
Transcriptome analysis of *Pdx* and *Cdx* mutant amphioxus embryos and larvae. **a** MA plot showing fold change in expression (log 2 scale) between wild type and *Pdx* mutant larvae in relation to mean expression level (read counts). Coloured dots are contigs meeting significance criteria. **b** MA plot showing fold change in expression (log 2 scale) between wild type and *Cdx* mutant embryos in relation to mean expression level (read counts). Coloured dots are contigs meeting significance criteria. **c** Violin plot comparing expression level (FPKM) between wild type (WT) and *Pdx* mutant larvae (KO) for selected *GFP*, *ILP1* and *ILPR* contigs, showing downregulation of *GFP* and upregulation of *ILPR* in mutants. **d** Violin plot comparing expression level between wild type (WT) and *Cdx* mutant embryos (KO) for selected *iLBP* contigs, showing downregulation in mutants. **e** Linear regression showing colinear-like response in expression level fold change of Hox genes to *Cdx* mutation. **f** Proportional Venn diagram showing overlap between contigs classed as gut-enriched (blue) and those expressed differentially after *Pdx* mutation (orange) or *Cdx* mutation (green)

To examine the downstream molecular consequences of *Pdx* and *Cdx* mutation, we sequenced transcriptomes of mutant larvae soon after morphological phenotypes are evident: 5 gill slits for *Pdx*^*−/−*^ and 2–3 gill slits (34 h and 42 h) for *Cdx*^*−/−*^ (42 h described below; mixed analysis in Additional file 2: Supplementary Data). Comparisons were made to transcriptome data from mixed wild type and heterozygous siblings to identify direct and indirect targets of *Cdx* and *Pdx* that are up- or downregulated after mutation (Fig. 6a, b; Additional file 1: Fig. S17, S18).

We first asked which of the multiple amphioxus GFP-encoding genes [62, 63] are affected in *Pdx* mutants. We found 11 contigs encoding GFP that were significantly downregulated (1.8 to 8.3 fold); 10 derive from tandemly duplicated amphioxus *GFP* genes that cannot be distinguished using short read data: *GFP-8*, *GFP-10*, *GFP-12* and *GFP-13* [63] (Additional file 1: Table S5). We conclude that one or more of these closely related *GFP* genes were affected when regionalisation of the gut was disrupted by *Pdx* mutation (Fig. 6c).

Second, we asked if signalling pathways were disrupted by ParaHox gene mutation. In *Pdx*^−/−^ mutants, we detect significant changes to expression of genes encoding components of the insulin-signalling pathway (~ 1.6 fold downregulation of two *IGFBP* contigs; 1.4 fold upregulation of *ILPR* [64]), but not the gut-expressed *Ilp1* gene itself (Fig. 6c; Additional file 1: Table S6). In *Cdx*^−/−^ mutants, we found downregulation of intracellular lipid-binding protein (*iLBP*) genes, which in vertebrates includes *CRABP* (cellular retinoic acid-binding protein), *CRBP* (cellular retinol-binding proteins) and *FABP* (fatty acid-binding proteins). In amphioxus, these have undergone extensive independent duplications [56, 65]. The contigs affected by *Cdx* mutation correspond to amphioxus *iLBP4* (formerly called *CRABP* [66]; 4.2 to 13.5 fold downregulation) and *iLBP6* (1.6 fold downregulation; Fig. 6d; Additional file 1: Table S7). The biochemical activities of these genes are unclear; it is possible that one or both encode proteins that bind RA, inhibiting the RA pathway [56]. Downregulation of *iLBP* genes in *Cdx* mutants suggests a second possible mechanism through which *Cdx* suppresses RA activity in the posterior of amphioxus: through positive regulation of *iLBP*s. Consistent with disrupted tail formation, which persists through development, we find the *Rootletin* gene is downregulated in the transcriptome of *Cdx* mutants (Additional file 1: Table S7). We detect no significant change to expression levels of *FGF* or *Wnt* genes, RAR, RXR or RALDH (Additional file 1; Additional file 2: Supplementary Data), but an increase in expression of *Brachyury-2* in *Cdx* mutants (Additional file 1: Table S7).

Third, we examined the effect of amphioxus *Cdx* mutation on Hox genes because in vertebrates the role of *Cdx* genes in body axis elongation is mediated, at least in part, through activation of central and some posterior Hox genes [18, 43, 67, 68]. Vertebrate *Cdx* genes may also have repressive effects on the most anterior Hox genes giving a colinear-like response: in *Xenopus tropicalis*, perturbation of *Cdx* gene activity caused the upregulation of paralogy groups 1 and 2 Hox genes and downregulation of paralogy groups 5 to 10/11 [19]. In amphioxus, Hox genes differ greatly in expression intensity in normal embryos [69–71] (Additional file 1: Table S8, Fig. S19, S20). Despite these quantitative differences, we detect a significant colinear-like response of Hox genes to amphioxus *Cdx* gene activity. In amphioxus *Cdx*^−/−^ mutants, *Hox-1* expression is upregulated (1.4 fold); *Hox2*, *Hox3* and *Hox4* are unaffected; *Hox5* and *Hox6* are mildly downregulated; and *Hox7* is strongly downregulated, although not each change is significant when genes are analysed individually (Fig. 6e; Additional file 1: Table S8, Fig. S21). This colinear-like response is consistent with amphioxus *Cdx* in normal development activating central and posterior Hox genes and repressing anterior Hox genes as part of an axis patterning system.

### *Pdx* and *Cdx* mutation affects gut-associated gene expression

To investigate further the association between *Pdx* and *Cdx* genes and the development of the gut, we tested whether gene sets affected by mutations include a high proportion of ‘gut-enriched’ genes. We used published *B. lanceolatum* transcriptome data [69] to define 2083 gut-enriched genes, represented by 4705 contigs in our study. These are genes with far higher expression in level in the gut than in most other adult tissues. We find 482 are differentially expressed in *Pdx*^−/−^ mutants and 218 in *Cdx*^−/−^ mutants (37 in common; Additional file 2: Supplementary Data). This equates to 8.3% of the *Pdx* differentially expressed contigs and 15.3% of the *Cdx* differentially expressed contigs (Fig. 6f), a significant enrichment (Additional file 1: Fig. S22). Hence, mutation of *Pdx* or *Cdx* has a disproportionate and significant effect on the expression of gut-enriched genes.

We also used this dataset to investigate the nature of changes that occurred in the gut of *Pdx*^*−/−*^ mutants. In the set of 482 gut-enriched contigs with altered expression in mutants, we identified 218 annotated protein-coding genes (Additional file 2: Supplementary Data). These include homologues of vertebrate proteins with well-characterised roles in gut function, including the digestive enzyme chymotrypsin (1.5 fold downregulation), a mucin that coats epithelia (1.9 fold downregulation), a glycosidase enzyme used for starch digestion (1.5 fold upregulation) and a proton-coupled transporter for intestinal absorption of folates (1.5 fold upregulation); Additional file 1: Table S9.

## Discussion

The development of technologies for generating targeted mutations has great potential in comparative and evolutionary developmental biology. The most widely used technology is based on CRISPR/Cas9, but this has not yet been applied successfully to cephalochordates. In contrast, TALENs have been used to generate inherited mutations in *B. floridae* [48, 49]. The method is efficient and reproducible, although not straightforward in amphioxus because of the practical difficulties of rearing from egg to adult and long generation times (4 to 6 months for *B. floridae*; much longer for *B. lanceolatum*). Here we report the introduction of germline mutations into two ParaHox genes, *Pdx* and *Cdx*. Each produces a clear phenotype. We do not know if all are complete null mutations or if alternative start codon usage, perhaps at low frequency, generates a hypomorphic allele. This is a possibility for *Pdx* since the mutant phenotype is subtle and not evident at embryonic stages, even though *Pdx* has no detectable maternal RNA [1, 14, 69]. Despite this caveat, the mutations allow robust conclusions to be drawn about important roles of amphioxus *Pdx* and *Cdx* genes, although they may not reveal every function.

Descriptive work has previously suggested that *Pdx* and *Cdx* genes might have functions in specific regions of the gut, notably the midgut for *Pdx* and the posterior gut or anus for *Cdx*. One of the earliest regional markers in amphioxus gut is *Ilp1*, expressed from the pharyngeal region to the midgut from gastrula to neurula, overlapping but larger than the *Pdx* domain [72, 73]. We found that mutation of amphioxus *Pdx* did not alter *Ilp1* transcript abundance in neurulae or larvae. Even if *Pdx* does not specify the *Ilp1* domain in early embryos, this does not exclude the possibility of *Pdx* being a transcriptional regulator of the *Ilp1* gene at later stages or in adults, comparable to *Pdx* regulation of *insulin* in the vertebrate pancreas [74]. A later regional marker in amphioxus endoderm is a stripe of endogenous green fluorescence, evident in the midgut from the 5 gill slit stage to larvae with many gill slits (around 46 days of development). Confocal imaging confirmed this is not fluorescence from ingested algae as it is present inside endoderm cells. Previous studies involving UV excitation of amphioxus larvae and adults have described green fluorescence primarily in ovaries and buccal cirri [62, 75] attributed to the presence of multiple *GFP* genes [62, 63]. To our knowledge, the stripe in larval gut has not been described previously, and it proved to be a fortuitous marker of endoderm regionalisation. The most striking phenotype we observed in *Pdx*^−/−^ mutant amphioxus was the absence of the GFP stripe. Transcriptome analysis reveals the endogenous gut GFP is encoded by one or more of an array of very similar genes (*GFP-8*, *GFP-10, GFP-12*, *GFP-13*). We interpret loss of the GFP stripe, and lower abundance of transcripts, as the consequence of incorrect cell fate specification in a region of endoderm. An alternative hypothesis, that the phenotype reflects an effect on *GFP* gene expression only, is not compatible with the large effect on the transcriptome we detect in *Pdx*^−/−^ mutants, including altered expression of genes encoding digestive enzymes and other gut markers. Since overall shape and size of the gut seems normal, we suggest that *Pdx* mutation has caused cells in one region of the gut to be transformed to another endodermal cell type, invoking *Pdx* in cell determination functions.

A gut phenotype is also evident in amphioxus *Cdx* mutants, specifically the failure of the anus to open. Opening of the anus in amphioxus development involves rearrangement of cell junctions within epithelial cell layers; cells at the extreme posterior of the gut must lessen connections with neighbours and form new junctions with outer epidermal cells. In this way, two nested epithelial cell layers (gut and epidermis) become contiguous, with the edges of the anus opening marking the boundary where the two layers fused. In terms of geometry, this marks a topological transition from an indented sphere to a torus [76]. Biologically, the propensity to break and reform epithelial cell junctions must be a property specific to the extreme posterior cells of the gut (and/or the epidermis). Hence, loss of this property in *Cdx* mutant larvae likely reflects a change in cell fate specification, with terminal gut cells losing a region-specific character.

Transcriptome analysis allowed further insight into gut-related functions. We found that a set of ‘gut-enriched’ genes was disproportionately represented among the (up and down) differentially regulated genes in both *Pdx* and *Cdx* mutant animals; for *Pdx*, these include genes encoding homologues of vertebrate digestive proteins. The true number of gut-associated genes affected is likely to be even higher, since our definition of ‘gut-enriched’ is relatively strict. This enrichment further indicates that correct development of the gut, with functional regionalisation, is severely perturbed in *Pdx* and *Cdx* mutants.

We also asked if amphioxus *Pdx* and *Cdx* genes have roles in tissues outside the gut, because *Pdx* is also expressed in the first Hesse organ receptor cells to form and *Cdx* is expressed in all germ layers at the posterior of the embryo [1]. Hesse organ receptors are primary rhabdomeric photoreceptors and express the amphioxus *melanopsin* (*Mop*) gene encoding the microvillar light transducing protein [77, 78]. We find that mutation of the *Pdx* gene did not remove *Mop* expression from Hesse organ receptor cells and the associated *Mitf*-positive pigment cell also formed. As in *Xenopus* [19], we find that disruption of *Cdx* function in amphioxus has a colinear-like effect on Hox genes. Morphological impacts of such change, such as homeotic transformations, are difficult to detect because of the lack of overt anatomical differences along the segmented mesoderm. We do detect cell fate change in the posterior epidermis in *Cdx* mutants, however, manifest as a smaller region of the epidermis expressing the *Rootletin* gene. The protein product of this gene drives cell shape changes underpinning tail fin development [50–52]; hence, this change in epidermal cell fate has a direct effect on larval morphology.

The other major developmental process in which *Cdx* genes are implicated in a range of taxa is posterior growth of the body or axial elongation. This role for *Cdx* is well characterised in vertebrates, and a similar function is evident in some arthropods, although homology of the process between distant taxa is not proven. We found that mutation of amphioxus *Cdx* dramatically disrupts growth of the body axis, as in vertebrates and some arthropods. We also detect some mechanistic similarities to vertebrates. The most important effectors through which *Cdx* genes control tail extension in vertebrates are central Hox genes and the retinoic acid (RA), FGF and Wnt signalling pathways [57, 58, 67, 68]. The role of vertebrate Hox genes in axial extension is complex. Although mouse Hox gene mutants do not generally have a tail phenotype (apart from *Hoxb13* [79]), ectopic expression of central genes, such as *Hoxa5* or *Hoxb8*, can partially rescue the tail truncation phenotype of *Cdx* mutants [68]. An opposite effect was found for *Hoxa13*, *Hoxb13* and *Hoxc13* suggesting a role for the extreme posterior Hox genes in tail growth termination [68, 80]. Furthermore, there is evidence that *Cdx2* is a direct activator of posterior and central Hox genes and these likely stimulate axial growth [41–43]. In amphioxus, we do not have definitive evidence for Hox gene involvement in tail development, but our finding of activation of central Hox genes by *Cdx* is consistent with this possibility and comparable to vertebrates.

There are clear similarities between amphioxus and vertebrate *Cdx* genes with respect to RA signalling in the tail. Low posterior levels of RA are necessary for tail growth in vertebrates [81–83] and amphioxus [51]. We show that *Cdx* is upstream of RA activity in amphioxus, as it is in vertebrates, since inhibition of RA action partially rescues the axial truncation and reduced tail fin phenotype of *Cdx* mutants. Young et al. [68] found that mutation of mouse *Cdx2* and *Cdx4* genes causes a downregulation of the gene encoding an RA-clearing enzyme *Cyp26A1*. Savory et al. [57] found this is a direct transcriptional effect and that it impacts RA signalling. Similarly, we find downregulation of expression of a *Cyp26* gene in amphioxus *Cdx* mutants and show this is also likely to be a direct transcriptional effect. The clear inference is that in both mice and amphioxus, *Cdx* genes suppress RA signalling in the tail through the same mechanism, activation of *Cyp26* expression, thereby permitting axis extension. There may be a second route by which amphioxus *Cdx* dampens posterior RA signalling, through positive regulation of genes encoding putative RA-binding proteins (*iLBP4* and *iLBP6*), although currently it is unclear if these proteins bind RA or another molecule. We also note differences to vertebrates. In particular, we do not detect altered expression of genes encoding FGF or Wnt signalling components in amphioxus *Cdx* mutants. This is consistent with the findings of Bertrand et al. [84], who showed that in amphioxus (unlike vertebrates) FGF does not act as an antagonist to RA signalling during posterior axial extension.

## Conclusions

We conclude that *Pdx* and *Cdx* genes are essential for regional cell fate specification in the endoderm of the developing amphioxus gut, including specification of midgut and formation of an anus. Combining with data from other taxa, specification of cell fates in the middle and posterior of the gut has probably been a function of *Pdx* and *Cdx* genes since the origin of Bilateria. This conclusion is consistent with the proposal that these two ParaHox genes played a role in the evolution of a through-gut, an innovation of the bilaterians and a possible contributor to sedimentary mixing and animal diversification in the Cambrian [1, 20]. We also conclude that a role for *Cdx* in posterior axial extension is homologous between amphioxus and vertebrates, with similar mechanistic basis, and thus dates back at least to the base of Chordata. This second role for *Cdx* may have facilitated the evolution of the extended post-anal tail and active locomotion in chordates.

## Methods

### Amphioxus culture and targeted mutation

Amphioxus (*Branchiostoma floridae*) were obtained from a stock maintained by Jr-Kai Yu originating from Tampa, Florida. Cultures were maintained in Xiamen University under previously described conditions [85]. Gametes were obtained using thermal-shock (20 to 26 °C) [86]; fertilisation and culturing of embryos at 26 °C was carried out as described [87]. The TALEN method was used to generate *Pdx* and *Cdx* mutants with TALEN pairs designed to target coding sequence (Additional file 1: Fig. S1-S6). TALEN construct assembly, mRNA sythesis and mutation efficacy assays were conducted following published methods [48]. Mosaic founder animals were spawned to generate F1 heterozygotes, using PCR and sequencing to detect, characterise and follow mutant alleles [49] (Additional file 1: Table S1). Homozygous mutants were generated by crossing heterozygous animals.

### Whole-mount in situ hybridisation (WMISH)

Coding sequences of *Cdx*, *Pdx*, *Rootletin*, *Ilp1*, *Mop*, *Mitf* and *Cyp26-3* genes were amplified from cDNA libraries from amphioxus embryos using primers given in Additional file 1: Table S2. PCR products were cloned into the pGEM-T Easy vector (Promega, USA) and confirmed by DNA sequencing; the resultant plasmids were linearised, cleaned using phenol-chloroform and used for templated synthesis of digoxigenin-labelled antisense RNA probes using T7 or SP6 RNA polymerase (Promega, USA). Embryos and larvae were fixed in 4% paraformaldehyde in MOPS buffer at 4 °C for 24 h and stored in 70% ethanol at − 20 °C. Hybridisation and detection was performed as described [88]; genotypes were checked by PCR after hybridisation and imaging.

### Scanning electron microscopy (SEM) and GFP detection

SEM used an adaptation of published methods [89]. In brief, embryos were fixed in 2.5% glutaraldehyde in PBS at 4 °C overnight, washed 3 × 10 min in 0.1 M PBS (pH 7.4) and transferred to 100% ethanol through a graded ethanol series. Specimens were then washed 5 × 10 min in 100% tertiary butyl alcohol and stored at 4 °C overnight. Samples were dried in a vacuum freeze dryer, placed on conductive tape, sprayed with platinum and observed under JSM-6390 scanning electron microscope. For GFP detection, amphioxus larvae were mounted in 1% methylcellulose in seawater and photographed under an SZX10 fluorescent stereoscope (Olympus, Japan) or an LSM 780 confocal microscope (Zeiss, Germany).

### BMS493 treatment

The retinoic acid antagonist BMS493 (Sigma Aldrich) was dissolved in DMSO as a 1 mM stock solution. Stock solution was diluted in filtered seawater to 1 μM and applied to amphioxus embryos from 5 h post-fertilisation onwards (early gastrula stage). Control embryos were treated with filtered seawater containing an equal amount of DMSO. Most embryos were washed and fixed at 16 or 18 h post-fertilisation for in situ hybridisation; others were continuously cultured in BMS493 until 30 h post-fertilisation for morphological observation.

### Reporter gene assays

A 3.1-kb region 5′ of *Cyp26-3* was cloned upstream of the firefly luciferase gene in pGL3 (Promega) and modified by PCR to generate three mutant versions with altered Cdx-binding sites (Additional file 1: Table S3). Solutions containing 3 ng/μL *Renilla* luciferase vector pRL-TK (Promega), 20% glycerol, 5 mg/ml Texas Red Dextran, with or without 30 ng/μL of one of the *Cyp26-3* constructs, were microinjected into unfertilised *B. floridae* eggs as previously described [87]. For each experiment, ~ 60 embryos were collected and assayed at 16 h post-fertilisation; uninjected embryos from the same batch were used as negative control. Levels of firefly luciferase from pGL3 and *Renilla* luciferase from pRL-TK were detected with the Dual Luciferase Kit (Promega) using a GloMax luminometer with an integration of 10 s; the level of firefly luciferase was normalised to *Renilla* luciferase activity for each experiment. All experiments were repeated three times (Additional file 1: Table S4).

### Embryo genotyping

Genotyping of live embryos was performed as previously described [49], and genotyping of embryos fixed with 4% PFA-MOPS-EGTA or analysed by whole-mount in situ hybridisation was conducted with the same protocol except that an extra 30-min wash in 500 ml filtered seawater or PBS was added before lysis, to remove the fixative.

### RNA sequencing

Embryos and larvae obtained from crosses between *Pdx*^*+/−*^ 4Δ × *Pdx*^*+/−*^ 11Δ and *Cdx*^*+/−*^ 7Δ × *Cdx*^*+/−*^ 7Δ were sorted by visual phenotype at the 5 gill slit stage for *Pdx* and 2–3 gill slits (34 h or 42 h post-fertilisation) for *Cdx*. Samples for RNA analysis were pools of ~ 100 (*Pdx*) or ~ 300 (*Cdx*) embryos or larvae. Each cross gave two matched sibling pools: ‘mutant’ individuals with a morphological phenotype (inferred *Pdx* 4Δ/11Δ and *Cdx* 7Δ/7Δ) and ‘control’ individuals without clear phenotype (mixture of heterozygotes and wild type). RNA sequencing was performed by BGI (Shenzhen, China) on the BGISEQ platform, > 60 million 100 nt paired-end reads per sample, using mRNA enrichment, random priming reverse transcription and low PCR cycle number; initial total RNA ranged from 1.11 to 2.17 μg (RIN 10.0) for *Pdx* and 0.69 to 1.89 μg (RIN 8.7 to 10) for *Cdx*. Reads were mapped using STAR v2.7.0 [90] using --twopassmode Basic and --outSJfilterOverhangMin 12 12 12 12 options [91] to *B. floridae* superTranscriptome BfsuperV5 (215,490 superTranscripts) downloaded from the IncDNA-BF database [92]; genes may be represented by more than one contig. Hox genes were assembled manually to remove and correct an artefactual fusion between 6 distinct Hox genes and a lncRNA (giving 215,495 superTranscripts [93];). Between 45.2 and 52.4 million reads per sample were mapped. Since superTranscripts can join exons not normally spliced together, STAR aligner settings were adjusted to permit mapping near non-canonical splice junctions (STAR --quantMode GeneCounts --twopassMode Basic --outSJfilterOverhangMin 12 12 12 12). STAR mapped 91% and 90% of reads from the *Pdx* and *Cdx* experiments. Reads were quantified using featureCounts [94] in the Subread package v1.6.3 allowing multiple mapping, thereby permitting analysis of duplicate genes (featureCounts -O –M -p –B –fraction).

### Differential gene expression analysis

Analysis of differentially expressed genes (DEGs) used DESeq2 v3.8 [95] including principal component analysis to test for outlier samples and batch effects. In the *Cdx* experiment, two control and two mutant 42-h samples grouped distinctly from one control and one mutant 34 h sample, likely due to a batch or developmental age effect. Two DEG analyses were therefore performed, 42 h alone and 34 h/42 h combined (Additional file 1: Fig. S17). In the *Pdx* experiment, one control sample (WT2) grouped distinctly and was excluded from DEG analysis which used 3 mutant and 2 control samples (Additional file 1: Fig. S18). For a contig to be considered differentially expressed, we required expression change of > 0.5 log_2_ (fold change) and adjusted *p* value < 0.05, plus absolute expression level of > 2 fpkm in at least one condition [19]. To assess the accuracy of embryo sorting, raw reads matching mutant or wild type allele sequences were counted. Embryo pools classed as *Cdx*^−/−^ had 4.3 to 5.3% of *Cdx* ‘wild type’ reads, suggesting that 1 or 2 embryos in each mutant pool of 20 were heterozygous; the control pool had 38 to 70% wild type reads, consistent with a mix of heterozygous and genetic wild type embryos. The same method could not be applied accurately to *Pdx* mutants because control pools had predominantly wild type reads (91 to 100%), suggesting downregulation of mutant allele expression in heterozygotes. This obviates the applicability of read counts for assessing heterozygote number. Gut-enriched genes were identified from published *B. lanceolatum* data [69] (NCBI GEO GSE106430) as genes with higher mean expression level in the gut than any other adult tissue (eggs and embryos excluded) and expression level in the gut at least double that in 7 out of 8 other adult tissues; the 2083 genes were matched to contigs in the current study using blastn with an *e* value cut off of 1e−70 giving 4705 gut-enriched contigs.

## Supplementary information

**Additional file 1: Figs. S1-S22, Tables S1-S9.** Gene structures of amphioxus *Pdx* and *Cdx* genes (Fig. S1-S2). Generation of mutations in amphioxus *Pdx* and *Cdx* genes (Fig. S3-S9, Table S1). Gene cloning primers (Table S2). Morphology of *Pdx* and *Cdx* mutant amphioxus (Fig. S10-S13). Analysis of amphioxus *Cyp26* genes (Fig. S14-S16, Tables S3-S4). Differential Gene Expression analysis from transcriptome data (Fig. S17-S21, Tables S5-S8). Effect of mutation of amphioxus *Pdx* and *Cdx* on gut-expressed genes (Fig. S22, Table S9).

**Additional file 2: Supplementary Data.** Gene expression differences between mutant and wild type amphioxus inferred from mapping RNAseq reads to superTranscriptome. Contig identity, expression fold change, FPKM, raw read counts and DNA sequence are given for *Cdx* gene mutation analysis (42 h dataset; 34 h and 42 h datasets) and *Pdx* mutation analysis. Data underlying identification of gut-enriched genes in *B. lanceolatum*, inference of inferred *B. floridae* orthologues, presence in *Pdx* and *Cdx* differential gene expression datasets and putative gene identities.

## Data Availability

Mutant and wild type RNAseq datasets generated during the current study have been deposited in NCBI SRA (accessions SRX7351856 to SRX7351867) linked to BioProject PRJNA594548 [96]. The superTranscriptome used for mapping, modified from IncDNA-BF, has been deposited in ORA-Data [93]. The DNA sequences of all contigs passing thresholds for inclusion in analysis, read mapping counts and RPKM values for each experiment are given in Additional file 2: Supplementary Data.
