# Supplementary material for: Mutation of amphioxus Pdx and Cdx demonstrates conserved roles for ParaHox genes in gut, anus and tail patterning
Source: BMC Biol. 2020 Jun 16;18:68. doi: 10.1186/s12915-020-00796-2 (PMC7296684; doi:10.1186/s12915-020-00796-2)
Supplement: Supplementary file 1 — Additional file 1: Figs. S1-S22, Tables S1-S9. Gene structures of amphioxus Pdx and Cdx genes (Fig. S1-S2). Generation of mutations in amphioxus Pdx and Cdx genes (Fig. S3-S9, Table S1). Gene cloning primers (Table S2). Morphology of Pdx and Cdx mutant amphioxus (Fig. S10-S13). Analysis of amphioxus Cyp26 genes (Fig. S14-S16, Tables S3-S4). Differential Gene Expression analysis from transcriptome data (Fig. S17-S21, Tables S5-S8). Effect of mutation of amphioxus Pdx and Cdx on gut-expressed genes (Fig. S22, Table S9). [file 12915_2020_796_MOESM1_ESM.pdf]

ADDITIONAL FILE 1

Mutation of amphioxus *Pdx* and *Cdx* demonstrates conserved roles for ParaHox genes in gut, anus and tail patterning

SECTION 1: GENE STRUCTURES OF AMPHIOXUS *PDX* AND *CDX* GENES

SECTION 2: GENERATION OF MUTATIONS IN AMPHIOUS *PDX* AND *CDX* GENES

SECTION 3: CLONING GENES FOR IN SITU HYBRIDISATION PROBES

SECTION 4: MORPHOLOGY OF *PDX* MUTANT AMPHIOXUS

SECTION 5: MORPHOLOGY OF *CDX* MUTANT AMPHIOXUS

SECTION 6: ANALYSIS OF *CYP26-3*

SECTION 7: DIFFERENTIAL EXPRESSION OF CANDIDATE TARGET GENES

SECTION 8: DIFFERENTIAL EXPRESSION OF GUT-ENRICHED GENES

SECTION 1: GENE STRUCTURES OF AMPHIOXUS *PDX* AND *CDX* GENES

(a) Verification of *Pdx* gene structure

The *Branchiostoma floridae Pdx (Xlox)* gene is comprised of two coding exons, confirmed here by aligning an assembled *Pdx* transcript sequence to the previously reported sequence of PAC clone 33B4 (GenBank AC129948.3; [2]); Figure S1.

<u>Complete predicted *B. floridae Pdx* open reading frame (homeodomain in **bold**)</u>

MIPASYQQHQARSSCLYANTQQPQHAMPYPPPNMSVVELDQLDAELPGGGMPGPGPMASSGPGPTQPVHH
AGPPPAPQSSCAVNRNENLPFPWMKTTKSHAHAWKSQWPGASFAVEDE**NKRTRTAYTRGQLLELEKEFHF**
NKYISRPRRIELAAMLNLTERHIKIWFQNRRMKWKKEQAKRRPLPESASSTTPGGSGGAGTAAGGAESTG
TSGTDPETSPVREPVSTPPASTSLPVSPPVNSGVQGTSAASHTGGVTVPPVHQTLPHSVTGPTEPTLQRE
NLSQSLAFSRS

<u>*Pdx* open reading frame</u>

ATGATCCCGGCGTCGTACCAGCAGCACCAGGCCCGGTCGTCCTGCCTGTACGCCAACACCCAACAGCCGC
AGCACGCCATGCCCTACCCGCCGCCAAACATGTCCGTCGTGGAGCTGGATCAGCTGGACGCAGAACTTCC
GGGCGGCGGCATGCCGGGGCCCGGCCCCATGGCGTCCTCGGGACCAGGCCCGACCCAGCCCGTCCACCAC
GCCGGCCCGCCCCCGGCCCCgCAGTCCAGcTGTGCCGTCAACAGGAACGAGAACCTGCCGTTCCCCTGGA
TGAAGACCACCAAGTCGCAcGCTCACGCCTGGAAGTCTCAGTGGCCAGGTGCGTCCTTCGCTGTTGAGGA
TGAGAACAAGAGAACGCGCACAGCCTACACCCGtGGCCAGCTCCTGGAGCTGGAGAAGGAGTTTCACTTC
AACAAGTACATTTCCCGGCCGCGCAGGATAGAGCTAGCCGCCATGCTCAACCTCACAGAGAGACACATCA
AAATCTGGTTCCAGAACCGCCGCATGAAGTGGAAAAAGGAGCAGGCaAAGCGGCGGCCGCTGCCCGAGTC
TGCCTCCAGCACGACCCCCGGGGGCAGCgGcGGGgCCGGCACCGCGGCGGGGGGCGCCGAGTCCACGGGG
ACCAGCGGCACCGACCCCGAGACTTCACCGGTCAGAGAGCCGGTCTCGACGCCTCCCGCCTCcACGTCTT
TACCGGTGTCTCCACCTGTGAACTCAGGTGTACAGGGGACCTCAGCAGCTTCCCACACGGGCGGGGTTAC
CGTTCCCCCCGTGCACCAAACACTGCCTCATAGCGTTACCGGACCGACAGAGCCCACACTCCAACGGGAA
AACCTCTCACAGAGCCTGGCCTTTTCACGCTCCTGA

<u>Exon structure in BAC clone 33B4</u>

49472bp–49798bp
MIPASYQQHQARSSCLYANTQQPQHAMPYPPPNMSVVELDQLDAELPGGGMPGPGPMASSGPGPTQPVHH
AGPPPAPQSSCAVNRNENLPFPWMKTTKSHAHAWKSQWP

57573bp-58118bp
GASFAVEDE**NKRTRTAYTRGQLLELEKEFHFNKYISRPRRIELAAMLNLTERHIKIWFQNRRMKWKKEQ**A
KRRPLPESASSTTPGGSGGAGTAAGGAESTGTSGTDPETSPVREPVSTPPASTSLPVSPPVNSGVQGTSA
ASHTGGVTVPPVHQTLPHSVTGPTEPTLQRENLSQSLAFSRS

Figure S1: *B. floridae Pdx* open reading frame and gene structure.

(b) Verification of *Cdx* gene structure

The *B. floridae Cdx* gene is generally depicted as comprised of two coding exons. However, we assembled a *B. floridae Cdx* transcript that aligned to three regions of the sequenced genomic PAC clone 36D2 (NCBI GenBank AC129947.4; [2]); Figure S2. We propose the small second exon is used differentially and note it is also present in predicted isoforms X1 and X2 (GenBank XP_019625318.1 and XP_019625319.1), but not X3 (XP_019625325.1), of *B. belcheri Cdx*.

<u>Complete predicted *B. floridae Cdx* open reading frame (homeodomain in **bold**)</u>

MYRHPSQGSYNLNPYNYATAHPAYPAEYGQYQVPPAVNAGENLQQTAAAAAWQSAAAFGSHGAGQRPEEWD
GRGYNCTAGTGLTAGPTGSCTAFPGMDYPVPVGAIQANSPAVSGVTTNSTNSQRPQHSRNPYDWMRKSNYS
TSPPPVLSVRGMPPQGRKDGGRCEILGPDGKTRT**KDKYRVVYSDHQRLELEKEFYSNKYITIKRKVQLANE**
LGLSERQVKIWFQNRRAKQRKMAKRKELQHPGGQGGSDDGGGVMGEVSTLTVGPPPHQLTLNPSGVAASTL
SNPALPPSSSPLMTSAMTHAVTLPSCVPSS

<u>*Cdx* open reading frame</u>

ATGTACCGTCACCCCTCCCAGGGCAGCTACAACTTGAACCCGTACAACTACGCCACGGCGCACCCTGCCTA
CCCCGCGGAGTACGGACAGTACCAGGTCCCGCCTGCCGTCAACGCCGGCGAGAACCTACAGCAGACGGCCG
CCGCCGCCGCGTGGCAGTCCGCCGCAGCCTTCGGCTCGCACGGGGCCGGACAGAGGCCAGAGGAATGGGAC
GGTCGCGGGTACAACTGCACGGCGGGGACCGGGCTGACCGCCGGCCCGACCGGGTCCTGTACAGCCTTCCC
CGGGATGGACTACCCTGTCCCCGTCGGTGCCATCCAGGCCAACAGCCCTGCCGTGTCGGGAGTGACGACCA
ACTCTACCAACAGTCAGAGACCACAGCACAGCAGAAATCCGTACGACTGGATGAGGAAAAGCAACTACTCC
ACAAGTCCTCCCCCAGTGCTGTCCGTGCGAGGCATGCCGCCGCAGGGCAGAAAGGATGGCGGCAGATGTGA
GATTCTAGGCCCTGATGGTAAGACGAGGACGAAGGATAAGTACCGGGTGGTTTATTCCGACCATCAGCGCC
TGGAGCTGGAGAAGGAGTTCTACTCCAACAAGTACATCACCATCAAGAGGAAGGTTCAGCTGGCGAACGAA
CTGGGCCTGTCGGAGCGCCAGGTCAAGATCTGGTTCCAGAACAGGCGCGCCAAGCAGCGCAAGATGGCCAA
GCGGAAGGAGCTGCAGCATCCGGGCGGGCAGGGCGGGAGTGACGATGGGGGAGGGGTGATGGGAGAGGTGT
CCACACTCACGGTAGGCCCCCCACCCCACCAGCTCACCCTAAACCCCAGCGGCGTGGCGGCCTCCACCCTC
AGCAACCCCGCTCTCCCCCCGTCCTCCTCCCCTCTCATGACCAGCGCCATGACGCATGCAGTGACGTTGCC
GTCGTGTGTTCCTTCCTCGTGA

<u>Exon structure in BAC clone 36D2</u>

42769bp–42329bp
MYRHPSQGSYNLNPYNYATAHPAYPAEYGQYQVPPAVNAGENLQQTAAAAAWQSAAAFGSHGAGQRPEEWD
GRGYNCTAGTGLTAGPTGSCTAFPGMDYPVPVGAIQANSPAVSGVTTNSTNSQRPQHSRNPYDWMRKSNYS
TSPPP

32571bp-32467bp
VLSVRGMPPQGRKDGGRCEILGPD

31997bp–31569bp
GKTRT**KDKYRVVYSDHQRLELEKEFYSNKYITIKRKVQLANELGLSERQVKIWFQNRRAKQRKMA**KRKELQ
HPGGQGGSDDGGGVMGEVSTLTVGPPPHQLTLNPSGVAASTLSNPALPPSSSPLMTSAMTHAVTLPSCVPS
S

Figure S2: *B. floridae Cdx* open reading frame and gene structure.

SECTION 2: GENERATION OF MUTATIONS IN AMPHIOXUS *PDX* AND *CDX* GENES

TALEN sequences used to target exon 1 of *B. floridae Pdx* and *Cdx* genes. For mutagenesis using TALENS, two in vitro transcribed RNAs are injected for each gene; each mRNA includes a region of Repeat Variable Di-residues (RVDs) encoding a sequence specific DNA-binding peptide, coupled to the catalytic domain of *FokI* nuclease. If two RVDs flank a site of interest, dimerization activates *FokI* nuclease activity, introducing DNA breaks leading to deletion mutations. Figures S3 to S6 give the sequences of the four in vitro transcribed RNAs used, from the T3 RNA polymerase binding site to the restriction enzyme site used for plasmid linearization before in vitro transcription.

Pdx forward TALEN

AATTAACCCTCACTAAAGGGAAGCTTGCTTGTTCTTTTTGCAGAAGCTCAGAATAAACGCTCAACTTTGGCAGATCTAACTCGAGAAA
GATATTGTATATATCGTAACAATAGGAGGTTCAACAATGGCTTCCTCCCCTCCAAAGAAAAAGAGAAAGGTTAGTTGGAAGGACGCAA
GTGGTTGGTCTAGAGTGGATCTACGCACGCTCGGCTACAGTCAGCAGCAAGAGAAGATCAAACCGAAGGTGCGTTCGACAGTGGC
GCAGCACCACGAGGCACTGGTGGGCCATGGGTTTACACACGCGCACATCGTTGCGCTCAGCCAACACCCGGCAGCGTTAGGGACCGTC
GCTGTCACGTATCAGCACATAATCACGGCGTTGCCAGAGGCGACACACGAAGACATCGTTGGCGTCGGCAAACAGTGGTCCGGCGCAC
GCGCCCTGGAGGCCTTGCTCACGGATGCGGGGGAGTTGAGAGGTCCGCCGTTACAGTTGGACACAGGCCAACTTGTGAAGATTGCAAA
ACGTGGCGGCGTGACCGCAATGGAGGCAGTGCATGCATCGCGCAATGCACTGACGGGTGCCCCCCTGAACCTGACCCCGGACCAAGTG
GTGGCTATCGCCAGCAACGGTGGCGGCAAGCAAGCGCTCGAAACGGTGCAGCGGCTGTTGCCGGTGCTGTGCCAGGACCATGGCCTGA
CCCCGGACCAAGTGGTGGCTATCGCCAGCAACAATGGCGGCAAGCAAGCGCTCGAAACGGTGCAGCGGCTGTTGCCGGTGCTGTGCCA
GGACCATGGCCTGACTCCGGACCAAGTGGTGGCTATCGCCAGCCACGATGGCGGCAAGCAAGCGCTCGAAACGGTGCAGCGGCTGTTG
CCGGTGCTGTGCCAGGACCATGGCCTGACTCCGGACCAAGTGGTGGCTATCGCCAGCCACGATGGCGGCAAGCAAGCGCTCGAAACGG
TGCAGCGGCTGTTGCCGGTGCTGTGCCAGGACCATGGCCTGACTCCGGACCAAGTGGTGGCTATCGCCAGCCACGATGGCGGCAAGCA
AGCGCTCGAAACGGTGCAGCGGCTGTTGCCGGTGCTGTGCCAGGACCATGGCCTGACCCCGGACCAAGTGGTGGCTATCGCCAGCAAC
GGTGGCGGCAAGCAAGCGCTCGAAACGGTGCAGCGGCTGTTGCCGGTGCTGTGCCAGGACCATGGCCTGACCCCGGACCAAGTGGTGG
CTATCGCCAGCAACATTGGCGGCAAGCAAGCGCTCGAAACGGTGCAGCGGCTGTTGCCGGTGCTGTGCCAGGACCATGGCCTGACTCC
GGACCAAGTGGTGGCTATCGCCAGCCACGATGGCGGCAAGCAAGCGCTCGAAACGGTGCAGCGGCTGTTGCCGGTGCTGTGCCAGGAC
CATGGCCTGACTCCGGACCAAGTGGTGGCTATCGCCAGCCACGATGGCGGCAAGCAAGCGCTCGAAACGGTGCAGCGGCTGTTGCCGG
TGCTGTGCCAGGACCATGGCCTGACTCCGGACCAAGTGGTGGCTATCGCCAGCCACGATGGCGGCAAGCAAGCGCTCGAAACGGTGCA
GCGGCTGTTGCCGGTGCTGTGCCAGGACCATGGCCTGACCCCGGACCAAGTGGTGGCTATCGCCAGCAACAATGGCGGCAAGCAAGCG
CTCGAAACGGTGCAGCGGCTGTTGCCGGTGCTGTGCCAGGACCATGGCCTGACTCCGGACCAAGTGGTGGCTATCGCCAGCCACGATG
GCGGCAAGCAAGCGCTCGAAACGGTGCAGCGGCTGTTGCCGGTGCTGTGCCAGGACCATGGCCTGACTCCGGACCAAGTGGTGGCTAT
CGCCAGCCACGATGGCGGCAAGCAAGCGCTCGAAACGGTGCAGCGGCTGTTGCCGGTGCTGTGCCAGGACCATGGCCTGACCCCGGAC
CAAGTGGTGGCTATCGCCAGCAACAATGGCGGCAAGCAAGCGCTCGAAAGCATTGTGGCCCAGCTGAGCCGGCCTGATCCGGCGTTGG
CCGCGTTGACCAACGACCACCTCGTCGCCTTGGCCTGCCTCGGCGGACGTCCTGCCATGGATGCAGTGAAAAAGGGATTGCCGCACGC
GCCGGAATTGATCAGAAGAGTCAATCGCCGTATTGGCGAACGCACGTCCCATCGCGTTGCCTCTAGATCCCAGCTAGTGAAATCTGAA
TTGGAAGAGAAGAAATCTGAACTTAGACATAAATTGAAATATGTGCCACATGAATATATTGAATTGATTGAAATCGCAAGAAATTCAA
CTCAGGATAGAATCCTTGAAATGAAGGTGATGGAGTTCTTTATGAAGGTTTATGGTTATCGTGGTAAACATTTGGGTGGATCAAGGAA
ACCAGACGGAGCAATTTATACTGTCGGATCTCCTATTGATTACGGTGTGATCGTTGATACTAAGGCATATTCAGGAGGTTATAATCTT
CCAATTGGTCAAGCAGATGAAATGCAAAGATATGTCGAAGAGAATCAAACAAGAAACAAGCATATCAACCCTAATGAATGGTGGAAAG
TCTATCCATCTTCAGTAACAGAATTTAAGTTCTTGTTTGTGAGTGGTCATTTCAAAGGAAACTACAAAGCTCAGCTTACAAGATTGAA
TCATATCACTAATTGTAATGGAGCTGTTCTTAGTGTAGAAGAGCTTTTGATTGGTGGAGAAATGATTAAAGCTGGTACATTGACACTT
GAGGAAGTGAGAAGGAAATTTAATAACGGTGAGATAAACTTTTAATAGGCTAGTGACTGACTAGGATCTGGTTACCACTAAACCAGCC
TCAAGAACACCCGAATGGAGTCTCTAAGCTACATAATACCAACTTACACTTACAAAATGTTGTCCCCCAAAATGTAGCCATTCGTATC
TGCTCCTAATAAAAAGAAAGTTTCTTCACATTCTAAAAAAAAAAAAAAAAAAAAAAAAAAAAAACCCCCCCCCCCCCCCCCCCCCCCC
CCCCCCGCATGCCTGCAGGTCGACTAGGATCCCCGGGTACCGAGCTC

Figure S3: *Pdx* forward TALEN. Red highlight = T3 promoter; blue text = 5’ and 3’ untranslated regions; red text = open reading frame for TALEN peptide sequence; red underlined text = sequence encoding RVDs; green highlight = *Sac*I linearization site.

Pdx reverse TALEN

AATTAACCCTCACTAAAGGGAAGCTTGCTTGTTCTTTTTGCAGAAGCTCAGAATAAACGCTCAACTTTGGCAGATCTAACTCGAGAAA
GATATTGTATATATCGTAACAATAGGAGGTTCAACAATGGCTTCCTCCCCTCCAAAGAAAAAGAGAAAGGTTAGTTGGAAGGACGCAA
GTGGTTGGTCTAGAGTGGATCTACGCACGCTCGGCTACAGTCAGCAGCAGCAAGAGAAGATCAAACCGAAGGTGCGTTCGACAGTGGC
GCAGCACCACGAGGCACTGGTGGGCCATGGGTTTACACACGCGCACATCGTTGCGCTCAGCCAACACCCGGCAGCGTTAGGGACCGTC
GCTGTCACGTATCAGCACATAATCACGGCGTTGCCAGAGGCGACACACGAAGACATCGTTGGCGTCGGCAAACAGTGGTCCGGCGCAC
GCGCCCTGGAGGCCTTGCTCACGGATGCGGGGGAGTTGAGAGGTCCGCCGTTACAGTTGGACACAGGCCAACTTGTGAAGATTGCAAA
ACGTGGCGGCGTGACCGCAATGGAGGCAGTGCATGCATCGCGCAATGCACTGACGGGTGCCCCCCTGAACCTGACCCCGGACCAAGTG
GTGGCTATCGCCAGCAACAATGGCGGCAAGCAAGCGCTCGAAACGGTGCAGCGGCTGTTGCCGGTGCTGTGCCAGGACCATGGCCTGA
CTCCGGACCAAGTGGTGGCTATCGCCAGCCACGATGGCGGCAAGCAAGCGCTCGAAACGGTGCAGCGGCTGTTGCCGGTGCTGTGCCA
GGACCATGGCCTGACCCCGGACCAAGTGGTGGCTATCGCCAGCAACGGTGGCGGCAAGCAAGCGCTCGAAACGGTGCAGCGGCTGTTG
CCGGTGCTGTGCCAGGACCATGGCCTGACCCCGGACCAAGTGGTGGCTATCGCCAGCAACAATGGCGGCAAGCAAGCGCTCGAAACGG
TGCAGCGGCTGTTGCCGGTGCTGTGCCAGGACCATGGCCTGACCCCGGACCAAGTGGTGGCTATCGCCAGCAACATTGGCGGCAAGCA
AGCGCTCGAAACGGTGCAGCGGCTGTTGCCGGTGCTGTGCCAGGACCATGGCCTGACCCCGGACCAAGTGGTGGCTATCGCCAGCAAC
GGTGGCGGCAAGCAAGCGCTCGAAACGGTGCAGCGGCTGTTGCCGGTGCTGTGCCAGGACCATGGCCTGACTCCGGACCAAGTGGTGG
CTATCGCCAGCCACGATGGCGGCAAGCAAGCGCTCGAAACGGTGCAGCGGCTGTTGCCGGTGCTGTGCCAGGACCATGGCCTGACTCC
GGACCAAGTGGTGGCTATCGCCAGCCACGATGGCGGCAAGCAAGCGCTCGAAACGGTGCAGCGGCTGTTGCCGGTGCTGTGCCAGGAC
CATGGCCTGACCCCGGACCAAGTGGTGGCTATCGCCAGCAACATTGGCGGCAAGCAAGCGCTCGAAACGGTGCAGCGGCTGTTGCCGG
TGCTGTGCCAGGACCATGGCCTGACCCCGGACCAAGTGGTGGCTATCGCCAGCAACAATGGCGGCAAGCAAGCGCTCGAAACGGTGCA
GCGGCTGTTGCCGGTGCTGTGCCAGGACCATGGCCTGACCCCGGACCAAGTGGTGGCTATCGCCAGCCACGATGGCGGCAAGCAAGCG
CTCGAAACGGTGCAGCGGCTGTTGCCGGTGCTGTGCCAGGACCATGGCCTGACCCCGGACCAAGTGGTGGCTATCGCCAGCAACGGTG
GCGGCAAGCAAGCGCTCGAAACGGTGCAGCGGCTGTTGCCGGTGCTGTGCCAGGACCATGGCCTGACTCCGGACCAAGTGGTGGCTAT
CGCCAGCCACGATGGCGGCAAGCAAGCGCTCGAAACGGTGCAGCGGCTGTTGCCGGTGCTGTGCCAGGACCATGGCCTGACTCCGGAC
CAAGTGGTGGCTATCGCCAGCCACGATGGCGGCAAGCAAGCGCTCGAAACGGTGCAGCGGCTGTTGCCGGTGCTGTGCCAGGACCATG
GCCTGACCCCGGACCAAGTGGTGGCTATCGCCAGCAACATTGGCGGCAAGCAAGCGCTCGAAACGGTGCAGCGGCTGTTGCCGGTGCT
GTGCCAGGACCATGGCCTGACCCCGGACCAAGTGGTGGCTATCGCCAGCCACGATGGCGGCAAGCAAGCGCTCGAAAGCATTGTGGCC
CAGCTGAGCCGGCCTGATCCGGCGTTGGCCGCGTTGACCAACGACCACCTCGTCGCCTTGGCCTGCCTCGGCGGACGTCCTGCCATGG
ATGCAGTGAAAAAGGGATTGCCGCACGCGCCGGAATTGATCAGAAGAGTCAATCGCCGTATTGGCGAACGCACGTCCCATCGCGTTGC
CTCTAGATCCCAGCTAGTGAAATCTGAATTGGAAGAGAAGAAATCTGAACTTAGACATAAATTGAAATATGTGCCACATGAATATATT
GAATTGATTGAAATCGCAAGAAATTCAACTCAGGATAGAATCCTTGAAATGAAGGTGATGGAGTTCTTTATGAAGGTTTATGGTTATC
GTGGTAAACATTTGGGTGGATCAAGGAAACCAGACGGAGCAATTTATACTGTCGGATCTCCTATTGATTACGGTGTGATCGTTGATAC
TAAGGCATATTCAGGAGGTTATAATCTTCCAATTGGTCAAGCAGATGAAATGCAAAGATATGTCGAAGAGAATCAAACAAGAAACAAG
CATATCAACCCTAATGAATGGTGGAAAGTCTATCCATCTTCAGTAACAGAATTTAAGTTCTTGTTTGTGAGTGGTCATTTCAAAGGAA
ACTACAAAGCTCAGCTTACAAGATTGAATCATATCACTAATTGTAATGGAGCTGTTCTTAGTGTAGAAGAGCTTTTGATTGGTGGAGA
AATGATTAAAGCTGGTACATTGACACTTGAGGAAGTGAGAAGGAAATTTAATAACGGTGAGATAAACTTTTAATAGGCTAGTGACTGA
CTAGGATCTGGTTACCACTAAACCAGCCTCAAGAACACCCGAATGGAGTCTCTAAGCTACATAATACCAACTTACACTTACAAAATGT
TGTCCCCCAAAATGTAGCCATTCGTATCTGCTCCTAATAAAAAGAAAGTTTCTTCACATTCTAAAAAAAAAAAAAAAAAAAAAAAAAA
AAAACCCCCCCCCCCCCCCCCCCCCCCCCCCCCCGCATGCCTGCAGGTCGACTAGGATCCCCGGGTACCGAGCTC

Figure S4: *Pdx* reverse TALEN. Red highlight = T3 promoter; blue text = 5’ and 3’ untranslated regions; red text = open reading frame for TALEN peptide sequence; red underlined text = sequence encoding RVDs; green highlight = *Sac*I linearization site.

Cdx forward TALEN

AATTAACCCTCACTAAAGGGAAGCTTGCTTGTTCTTTTTGCAGAAGCTCAGAATAAACGCTCAACTTTGGCAGATCTAACTCGAGAAA
GATATTGTATATATCGTAACAATAGGAGGTTCAACAATGGCTTCCTCCCCTCCAAAGAAAAAGAGAAAGGTTAGTTGGAAGGACGCAA
GTGGTTGGTCTAGAGTGGATCTACGCACGCTCGGCTACAGTCAGCAGCAGCAAGAGAAGATCAAACCGAAGGTGCGTTCGACAGTGGC
GCAGCACCACGAGGCACTGGTGGGCCATGGGTTTACACACGCGCACATCGTTGCGCTCAGCCAACACCCGGCAGCGTTAGGGACCGTC
GCTGTCACGTATCAGCACATAATCACGGCGTTGCCAGAGGCGACACACGAAGACATCGTTGGCGTCGGCAAACAGTGGTCCGGCGCAC
GCGCCCTGGAGGCCTTGCTCACGGATGCGGGGGAGTTGAGAGGTCCGCCGTTACAGTTGGACACAGGCCAACTTGTGAAGATTGCAAA
ACGTGGCGGCGTGACCGCAATGGAGGCAGTGCATGCATCGCGCAATGCACTGACGGGTGCCCCCCTGAACCTGACCCCGGACCAAGTG
GTGGCTATCGCCAGCCACGATGGCGGCAAGCAAGCGCTCGAAACGGTGCAGCGGCTGTTGCCGGTGCTGTGCCAGGACCATGGCCTGA
CTCCGGACCAAGTGGTGGCTATCGCCAGCCACGATGGCGGCAAGCAAGCGCTCGAAACGGTGCAGCGGCTGTTGCCGGTGCTGTGCCA
GGACCATGGCCTGACCCCGGACCAAGTGGTGGCTATCGCCAGCAACAATGGCGGCAAGCAAGCGCTCGAAACGGTGCAGCGGCTGTTG
CCGGTGCTGTGCCAGGACCATGGCCTGACTCCGGACCAAGTGGTGGCTATCGCCAGCCACGATGGCGGCAAGCAAGCGCTCGAAACGG
TGCAGCGGCTGTTGCCGGTGCTGTGCCAGGACCATGGCCTGACTCCGGACCAAGTGGTGGCTATCGCCAGCCACGATGGCGGCAAGCA
AGCGCTCGAAACGGTGCAGCGGCTGTTGCCGGTGCTGTGCCAGGACCATGGCCTGACCCCGGACCAAGTGGTGGCTATCGCCAGCAAC
ATTGGCGGCAAGCAAGCGCTCGAAACGGTGCAGCGGCTGTTGCCGGTGCTGTGCCAGGACCATGGCCTGACCCCGGACCAAGTGGTGG
CTATCGCCAGCAACGGTGGCGGCAAGCAAGCGCTCGAAACGGTGCAGCGGCTGTTGCCGGTGCTGTGCCAGGACCATGGCCTGACCCC
GGACCAAGTGGTGGCTATCGCCAGCAACAATGGCGGCAAGCAAGCGCTCGAAACGGTGCAGCGGCTGTTGCCGGTGCTGTGCCAGGAC
CATGGCCTGACCCCGGACCAAGTGGTGGCTATCGCCAGCAACGGTGGCGGCAAGCAAGCGCTCGAAACGGTGCAGCGGCTGTTGCCGG
TGCTGTGCCAGGACCATGGCCTGACCCCGGACCAAGTGGTGGCTATCGCCAGCAACATTGGCGGCAAGCAAGCGCTCGAAACGGTGCA
GCGGCTGTTGCCGGTGCTGTGCCAGGACCATGGCCTGACCCCGGACCAAGTGGTGGCTATCGCCAGCCACGATGGCGGCAAGCAAGCG
CTCGAAACGGTGCAGCGGCTGTTGCCGGTGCTGTGCCAGGACCATGGCCTGACTCCGGACCAAGTGGTGGCTATCGCCAGCCACGATG
GCGGCAAGCAAGCGCTCGAAACGGTGCAGCGGCTGTTGCCGGTGCTGTGCCAGGACCATGGCCTGACCCCGGACCAAGTGGTGGCTAT
CGCCAGCAACAATGGCGGCAAGCAAGCGCTCGAAACGGTGCAGCGGCTGTTGCCGGTGCTGTGCCAGGACCATGGCCTGACCCCGGAC
CAAGTGGTGGCTATCGCCAGCAACGGTGGCGGCAAGCAAGCGCTCGAAACGGTGCAGCGGCTGTTGCCGGTGCTGTGCCAGGACCATG
GCCTGACTCCGGACCAAGTGGTGGCTATCGCCAGCCACGATGGCGGCAAGCAAGCGCTCGAAACGGTGCAGCGGCTGTTGCCGGTGCT
GTGCCAGGACCATGGCCTGACCCCGGACCAAGTGGTGGCTATCGCCAGCAACATTGGCGGCAAGCAAGCGCTCGAAAGCATTGTGGCC
CAGCTGAGCCGGCCTGATCCGGCGTTGGCCGCGTTGACCAACGACCACCTCGTCGCCTTGGCCTGCCTCGGCGGACGTCCTGCCATGG
ATGCAGTGAAAAAGGGATTGCCGCACGCGCCGGAATTGATCAGAAGAGTCAATCGCCGTATTGGCGAACGCACGTCCCATCGCGTTGC
CTCTAGATCCCAGCTAGTGAAATCTGAATTGGAAGAGAAGAAATCTGAACTTAGACATAAATTGAAATATGTGCCACATGAATATATT
GAATTGATTGAAATCGCAAGAAATTCAACTCAGGATAGAATCCTTGAAATGAAGGTGATGGAGTTCTTTATGAAGGTTTATGGTTATC
GTGGTAAACATTTGGGTGGATCAAGGAAACCAGACGGAGCAATTTATACTGTCGGATCTCCTATTGATTACGGTGTGATCGTTGATAC
TAAGGCATATTCAGGAGGTTATAATCTTCCAATTGGTCAAGCAGATGAAATGCAAAGATATGTCGAAGAGAATCAAACAAGAAACAAG
CATATCAACCCTAATGAATGGTGGAAAGTCTATCCATCTTCAGTAACAGAATTTAAGTTCTTGTTTGTGAGTGGTCATTTCAAAGGAA
ACTACAAAGCTCAGCTTACAAGATTGAATCATATCACTAATTGTAATGGAGCTGTTCTTAGTGTAGAAGAGCTTTTGATTGGTGGAGA
AATGATTAAAGCTGGTACATTGACACTTGAGGAAGTGAGAAGGAAATTTAATAACGGTGAGATAAACTTTTAATAGGCTAGTGACTGA
CTAGGATCTGGTTACCACTAAACCAGCCTCAAGAACACCCGAATGGAGTCTCTAAGCTACATAATACCAACTTACACTTACAAAATGT
TGTCCCCCAAAATGTAGCCATTCGTATCTGCTCCTAATAAAAAGAAAGTTTCTTCACATTCTAAAAAAAAAAAAAAAAAAAAAAAAAA
AAAACCCCCCCCCCCCCCCCCCCCCCCCCCCCCCGCATGCCTGCAGGTCGACTAGGATCCCCGGGTACCGAGCTC

Figure S5: *Cdx* forward TALEN. Red highlight = T3 promoter; blue text = 5’ and 3’ untranslated regions; red text = open reading frame for TALEN peptide sequence; red underlined text = sequence encoding RVDs; green highlight = *Sac*I linearization site.

Cdx reverse TALEN

AATTAACCCTCACTAAAGGGAAGCTTGCTTGTTCTTTTTGCAGAAGCTCAGAATAAACGCTCAACTTTGGCAGATCTAACTCGAGAAA
GATATTGTATATATCGTAACAATAGGAGGTTCAACAATGGCTTCCTCCCCTCCAAAGAAAAAGAGAAAGGTTAGTTGGAAGGACGCAA
GTGGTTGGTCTAGAGTGGATCTACGCACGCTCGGCTACAGTCAGCAGCAAGAGAAGATCAAACCGAAGGTGCGTTCGACAGTGGC
GCAGCACCACGAGGCACTGGTGGGCCATGGGTTTACACACGCGCACATCGTTGCGCTCAGCCAACACCCGGCAGCGTTAGGGACCGTC
GCTGTCACGTATCAGCACATAATCACGGCGTTGCCAGAGGCGACACACGAAGACATCGTTGGCGTCGGCAAACAGTGGTCCGGCGCAC
GCGCCCTGGAGGCCTTGCTCACGGATGCGGGGGAGTTGAGAGGTCCGCCGTTACAGTTGGACACAGGCCAACTTGTGAAGATTGCAAA
ACGTGGCGGCGTGACCGCAATGGAGGCAGTGCATGCATCGCGCAATGCACTGACGGGTGCCCCCCTGAACCTGACCCCGGACCAAGTG
GTGGCTATCGCCAGCAACATTGGCGGCAAGCAAGCGCTCGAAACGGTGCAGCGGCTGTTGCCGGTGCTGTGCCAGGACCATGGCCTGA
CTCCGGACCAAGTGGTGGCTATCGCCAGCCACGATGGCGGCAAGCAAGCGCTCGAAACGGTGCAGCGGCTGTTGCCGGTGCTGTGCCA
GGACCATGGCCTGACCCCGGACCAAGTGGTGGCTATCGCCAGCAACAATGGCGGCAAGCAAGCGCTCGAAACGGTGCAGCGGCTGTTG
CCGGTGCTGTGCCAGGACCATGGCCTGACCCCGGACCAAGTGGTGGCTATCGCCAGCAACAATGGCGGCAAGCAAGCGCTCGAAACGG
TGCAGCGGCTGTTGCCGGTGCTGTGCCAGGACCATGGCCTGACCCCGGACCAAGTGGTGGCTATCGCCAGCAACAATGGCGGCAAGCA
AGCGCTCGAAACGGTGCAGCGGCTGTTGCCGGTGCTGTGCCAGGACCATGGCCTGACCCCGGACCAAGTGGTGGCTATCGCCAGCAAC
GGTGGCGGCAAGCAAGCGCTCGAAACGGTGCAGCGGCTGTTGCCGGTGCTGTGCCAGGACCATGGCCTGACCCCGGACCAAGTGGTGG
CTATCGCCAGCAACGGTGGCGGCAAGCAAGCGCTCGAAACGGTGCAGCGGCTGTTGCCGGTGCTGTGCCAGGACCATGGCCTGACTCC
GGACCAAGTGGTGGCTATCGCCAGCCACGATGGCGGCAAGCAAGCGCTCGAAACGGTGCAGCGGCTGTTGCCGGTGCTGTGCCAGGAC
CATGGCCTGACCCCGGACCAAGTGGTGGCTATCGCCAGCAACATTGGCGGCAAGCAAGCGCTCGAAACGGTGCAGCGGCTGTTGCCGG
TGCTGTGCCAGGACCATGGCCTGACCCCGGACCAAGTGGTGGCTATCGCCAGCAACATTGGCGGCAAGCAAGCGCTCGAAACGGTGCA
GCGGCTGTTGCCGGTGCTGTGCCAGGACCATGGCCTGACCCCGGACCAAGTGGTGGCTATCGCCAGCAACAATGGCGGCAAGCAAGCG
CTCGAAACGGTGCAGCGGCTGTTGCCGGTGCTGTGCCAGGACCATGGCCTGACCCCGGACCAAGTGGTGGCTATCGCCAGCAACGGTG
GCGGCAAGCAAGCGCTCGAAACGGTGCAGCGGCTGTTGCCGGTGCTGTGCCAGGACCATGGCCTGACCCCGGACCAAGTGGTGGCTAT
CGCCAGCAACGGTGGCGGCAAGCAAGCGCTCGAAACGGTGCAGCGGCTGTTGCCGGTGCTGTGCCAGGACCATGGCCTGACCCCGGAC
CAAGTGGTGGCTATCGCCAGCAACAATGGCGGCAAGCAAGCGCTCGAAACGGTGCAGCGGCTGTTGCCGGTGCTGTGCCAGGACCATG
GCCTGACCCCGGACCAAGTGGTGGCTATCGCCAGCAACGGTGGCGGCAAGCAAGCGCTCGAAACGGTGCAGCGGCTGTTGCCGGTGCT
GTGCCAGGACCATGGCCTGACCCCGGACCAAGTGGTGGCTATCGCCAGCAACATTGGCGGCAAGCAAGCGCTCGAAAGCATTGTGGCC
CAGCTGAGCCGGCCTGATCCGGCGTTGGCCGCGTTGACCAACGACCACCTCGTCGCCTTGGCCTGCCTCGGCGGACGTCCTGCCATGG
ATGCAGTGAAAAAGGGATTGCCGCACGCGCCGGAATTGATCAGAAGAGTCAATCGCCGTATTGGCGAACGCACGTCCCATCGCGTTGC
CTCTAGATCCCAGCTAGTGAAATCTGAATTGGAAGAGAAGAAATCTGAACTTAGACATAAATTGAAATATGTGCCACATGAATATATT
GAATTGATTGAAATCGCAAGAAATTCAACTCAGGATAGAATCCTTGAAATGAAGGTGATGGAGTTCTTTATGAAGGTTTATGGTTATC
GTGGTAAACATTTGGGTGGATCAAGGAAACCAGACGGAGCAATTTATACTGTCGGATCTCCTATTGATTACGGTGTGATCGTTGATAC
TAAGGCATATTCAGGAGGTTATAATCTTCCAATTGGTCAAGCAGATGAAATGCAAAGATATGTCGAAGAGAATCAAACAAGAAACAAG
CATATCAACCCTAATGAATGGTGGAAAGTCTATCCATCTTCAGTAACAGAATTTAAGTTCTTGTTTGTGAGTGGTCATTTCAAAGGAA
ACTACAAAGCTCAGCTTACAAGATTGAATCATATCACTAATTGTAATGGAGCTGTTCTTAGTGTAGAAGAGCTTTTGATTGGTGGAGA
AATGATTAAAGCTGGTACATTGACACTTGAGGAAGTGAGAAGGAAATTTAATAACGGTGAGATAAACTTTTAATAGGCTAGTGACTGA
CTAGGATCTGGTTACCACTAAACCAGCCTCAAGAACACCCGAATGGAGTCTCTAAGCTACATAATACCAACTTACACTTACAAAATGT
TGTCCCCCAAAATGTAGCCATTCGTATCTGCTCCTAATAAAAAGAAAGTTTCTTCACATTCTAAAAAAAAAAAAAAAAAAAAAAAAAA
AAAACCCCCCCCCCCCCCCCCCCCCCCCCCCCCCGCATGCCTGCAGGTCGACTAGGATCCCCGGGTACCGAGCTC

Figure S6: *Cdx* reverse TALEN. Red highlight = T3 promoter; blue text = 5’ and 3’ untranslated regions; red text = open reading frame for TALEN peptide sequence; red underlined text = sequence encoding RVDs; green highlight = *Sac*I linearization site.

Each TALEN pair is designed to flank a restriction endonuclease recognition site enabling mutation detection by digestion of a PCR amplified product (*Afl*III for *Pdx*, *Pas*I for *Cdx*). Table 1 gives the mutation detection primers used.

**Genes**	**Primer sequences (5’→3’)**
Pdx mutation detection	Forward: TTTCAAACGATACCGGACAAAC<br>Reverse: CCACTGAGACTTCCAGGCGT
Cdx mutation detection	Forward: TACTGGTTTGTCACGGCGAG<br>Reverse: CTGGGGGAGGACTTGTGGAGTA

Table S1: Mutation detection primers

B. floridae eggs were injected with pairs of TALEN RNAs, fertilized and embryos reared to neurula stage, using previously described methods [48]. PCR followed by restriction digestion revealed that both TALEN pairs introduced deletion mutations, with a higher frequency detected by the Cdx TALEN pair (Figure S7).

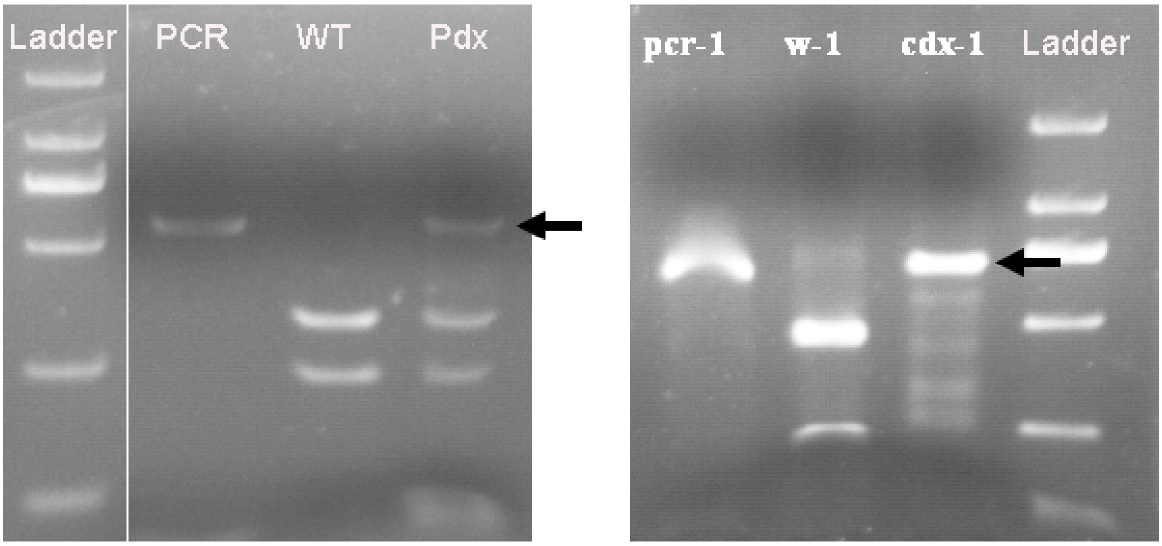

Figure S7: PCR analysis of embryos reared from injected eggs: Left hand gel: *Pdx* PCR products showing faint band of uncut amplification product (deletion mutants, arrow). Right hand gel: *Cdx* PCR products showing uncut amplification product (arrow).

The same PCR primers were used to track inheritance of the mutant alleles in adult amphioxus tail clips, in sperm or pools of embryos, and in single embryos after in situ hybridization, using previously described methods [48]. Sequencing of PCR products was also used to verify exact nature of the mutations.

The TALEN-generated *Pdx* mutants used in this study have deletions of 4 bp, 11 bp and 13 bp (Figure 1, main text; Figure S8). The predicted mutant peptides are shown beneath the DNA sequences; no predicted product contains a homeodomain.

- 4 bp *Pdx* deleted region (red) and predicted protein product

ATGATCCCGGCGTCGTACCAGCAGCACCAGGCCCGGTCGTCCTGCCTGTACGCCAACACCCAACAGCCGCAGC
ACGCCATGCCCTACCCGCCGCC**GCACAT**GTCCGTCGTGGAGCTGGATCAGCTGGACGCAGAACTTCCGGGCGG
CGGCATGCCGGGGCCCGGCCCCATGGCGTCCTCGGGACCAGGCCCGACCCAGCCCGTCCACCACGCCGGCCCG
CCCCCGGCCCCgCAGTCCAGcTGTGCCGTCAACAGGAACGAGAACCTGCCGTTCCCCTGGATGAAGACCACCA
AGTCGCAcGCTCACGCCTGGAAGTCTCAGTGGCCAGGTGCGTCCTTcGCTGTTGAGGATGAGAACAAGAGAAC
GCGCACAGCCTACACCCGtGGCCAGCTCCTGGAGCTGGAGAAGGAGTTTCACTTCAACAAGTACATTTCCCGG
CCGCGCAGGATAGAGCTAGCCGCCATGCTCAACCTCACAGAGAGACACATCAAAATCTGGTTCCAGAACCGCC
GCATGAAGTGGAAAAAGGAGCAGGCaAAGCGGCGGCCGCTGCCCGAGTCTGCCTCCAGCACGACCCCCGGGGG
CAGCgGcGGGgCCGGCACCGCGGCGGGGGGCGCCGAGTCCACGGGGACCAGCGGCACCGACCCCGAGACTTCA
CCGGTCAGAGAGCCGGTCTCGACGCCTCCCGCCTCcACGTCTTTACCGGTGTCTCCACCTGTGAACTCAGGTG
TACAGGGGACCTCAGCAGCTTCCCACACGGGCGGGGTTACCGTTCCCCCCGTGCACCAAACACTGCCTCATAG
CGTTACCGGACCGACAGAGCCCACACTCCAACGGGAAAACCTCTCACAGAGCCTGGCCTTTTCACGCTCCTGA

MIPASYQQHQARSSCLYANTQQPQHAMPYPPPRPSWSWISWTQNFRAAACRGPAPWRPRDQARPSPSTTPARP
RPRSPAVPSTGTRTCRSPG*

- 11 bp *Pdx* deleted region (red) and predicted protein product

ATGATCCCGGCGTCGTACCAGCAGCACCAGGCCCGGTCGTCCTGCCTGTACGCCAACACCCAACAGCCGCAGC
ACGCCATGCCCTACCCGCCGCCA**GC**C**ATGTCCGTCGT**GGAGCTGGATCAGCTGGACGCAGAACTTCCGGGCGG
CGGCATGCCGGGGCCCGGCCCCATGGCGTCCTCGGGACCAGGCCCGACCCAGCCCGTCCACCACGCCGGCCCG
CCCCCGGCCCCgCAGTCCAGcTGTGCCGTCAACAGGAACGAGAACCTGCCGTTCCCCTGGATGAAGACCACCA
AGTCGCAcGCTCACGCCTGGAAGTCTCAGTGGCCAGGTGCGTCCTTCGCTGTTGAGGATGAGAACAAGAGAAC
GCGCACAGCCTACACCCGtGGCCAGCTCCTGGAGCTGGAGAAGGAGTTTCACTTCAACAAGTACATTTCCCGG
CCGCGCAGGATAGAGCTAGCCGCCATGCTCAACCTCACAGAGAGACACATCAAAATCTGGTTCCAGAACCGCC
GCATGAAGTGGAAAAAGGAGCAGGCaAAGCGGCGGCCGCTGCCCGAGTCTGCCTCCAGCACGACCCCCGGGGG
CAGCgGcGGGgCCGGCACCGCGGCGGGGGGCGCCGAGTCCACGGGGACCAGCGGCACCGACCCCGAGACTTCA
CCGGTCAGAGAGCCGGTCTCGACGCCTCCCGCCTCcACGTCTTTACCGGTGTCTCCACCTGTGAACTCAGGTG
TACAGGGGACCTCAGCAGCTTCCCACACGGGCGGGGTTACCGTTCCCCCCGTGCACCAAACACTGCCTCATAG
CGTTACCGGACCGACAGAGCCCACACTCCAACGGGAAAACCTCTCACAGAGCCTGGCCTTTTCACGCTCCTGA

MIPASYQQHQARSSCLYANTQQPQHAMPYPPPAGAGSAGRRTSGRRHAGARPHGVLGTRPDPARPPRRPAPGP
AVQLCRQQEREPAVPLDEDHQVARSRLEVSVARCVLRC*

- 13 bp *Pdx* deleted region (red) and predicted protein product

ATGATCCCGGCGTCGTACCAGCAGCACCAGGCCCGGTCGTCCTGCCTGTACGCCAACACCCAACAGCCGCAGC
ACGCCATGCCCTACCC**GCCGCCAAACATG**TCCGTCGTGGAGCTGGATCAGCTGGACGCAGAACTTCCGGGCGG
CGGCATGCCGGGGCCCGGCCCCATGGCGTCCTCGGGACCAGGCCCGACCCAGCCCGTCCACCACGCCGGCCCG
CCCCCGGCCCCgCAGTCCAGcTGTGCCGTCAACAGGAACGAGAACCTGCCGTTCCCCTGGATGAAGACCACCA
AGTCGCAcGCTCACGCCTGGAAGTCTCAGTGGCCAGGTGCGTCCTTcGCTGTTGAGGATGAGAACAAGAGAAC
GCGCACAGCCTACACCCGtGGCCAGCTCCTGGAGCTGGAGAAGGAGTTTCACTTCAACAAGTACATTTCCCGG
CCGCGCAGGATAGAGCTAGCCGCCATGCTCAACCTCACAGAGAGACACATCAAAATCTGGTTCCAGAACCGCC
GCATGAAGTGGAAAAAGGAGCAGGCaAAGCGGCGGCCGCTGCCCGAGTCTGCCTCCAGCACGACCCCCGGGGG
CAGCgGcGGGgCCGGCACCGCGGCGGGGGGCGCCGAGTCCACGGGGACCAGCGGCACCGACCCCGAGACTTCA
CCGGTCAGAGAGCCGGTCTCGACGCCTCCCGCCTCcACGTCTTTACCGGTGTCTCCACCTGTGAACTCAGGTG
TACAGGGGACCTCAGCAGCTTCCCACACGGGCGGGGTTACCGTTCCCCCCGTGCACCAAACACTGCCTCATAG
CGTTACCGGACCGACAGAGCCCACACTCCAACGGGAAAACCTCTCACAGAGCCTGGCCTTTTCACGCTCCTGA

MIPASYQQHQARSSCLYANTQQPQHAMPYPPSWSWISWTQNFRAAACRGPAPWRPRDQARPSPSTTPARPRPR
SPAVPSTGTRTCRSPG*

Figure S8: Sites of 4 bp, 11 bp and 13 bp deletions in *Pdx* gene (red) and predicted protein products (out of frame amino acids underlined). For 4Δ and 11Δ we also identified substitution mutations generated next to the deletion (green). These are taken account of in predicting peptide sequence.

The TALEN-generated *Cdx* mutant used in this study has a deletion of 7 bp (Figure 1, main text; Figure S9). The predicted mutant peptide retains the first 5 amino acids before a frameshift, then 4 additional residues (underlined) before a premature stop codon.

<u>7 bp *Cdx* deleted region (red) and predicted protein product</u>

ATGTACCGTCACCC**CTCCCAG**GGCAGCTACAACTTGAACCCGTACAACTACGCCACGGCGCACCCTGCC
TACCCCGCGGAGTACGGACAGTACCAGGTCCCGCCTGCCGTCAACGCCGGCGAGAACCTACAGCAGACG
GCCGCCGCCGCCGCGTGGCAGTCCGCCGCAGCCTTCGGCTCGCACGGGGCCGGACAGAGGCCAGAGGAA
TGGGACGGTCGCGGGTACAACTGCACGGCGGGGACCGGGCTGACCGCCGGCCCGACCGGGTCCTGTACA
GCCTTCCCCGGGATGGACTACCCTGTCCCCGTCGGTGCCATCCAGGCCAACAGCCCTGCCGTGTCGGGA
GTGACGACCAACTCTACCAACAGTCAGAGACCACAGCACAGCAGAAATCCGTACGACTGGATGAGGAAA
AGCAACTACTCCACAAGTCCTCCCCCAGTGCTGTCCGTGCGAGGCATGCCGCCGCAGGGCAGAAAGGAT
GGCGGCAGATGTGAGATTCTAGGCCCTGATGGTAAGACGAGGACGAAGGATAAGTACCGGGTGGTTTAT
TCCGACCATCAGCGCCTGGAGCTGGAGAAGGAGTTCTACTCCAACAAGTACATCACCATCAAGAGGAAG
GTTCAGCTGGCGAACGAACTGGGCCTGTCGGAGCGCCAGGTCAAGATCTGGTTCCAGAACAGGCGCGCC
AAGCAGCGCAAGATGGCCAAGCGGAAGGAGCTGCAGCATCCGGGCGGGCAGGGCGGGAGTGACGATGGG
GGAGGGGTGATGGGAGAGGTGTCCACACTCACGGTAGGCCCCCCACCCCACCAGCTCACCCTAAACCCC
AGCGGCGTGGCGGCCTCCACCCTCAGCAACCCCGCTCTCCCCCCGTCCTCCTCCCCTCTCATGACCAGC
GCCATGACGCATGCAGTGACGTTGCCGTCGTGTGTTCCTTCCTCGTGA

MYRHP<u>AATT</u>*

Figure S9: Site of 7 bp deletion in *Cdx* gene (red) and predicted protein product (out of frame amino acids underlined)

SECTION 3: CLONING GENES FOR IN SITU HYBRIDISATION PROBES

Genes	Primer sequences (5'→3')
Pdx	Forward: GGTACCTACCCACGAGAAGGGTACGA<br>Reverse: GAATTCGGAGAGCCGTTGTTGACGTA
Cdx	Forward: ATGTACCGTCACCCCTCCCAGGG<br>Reverse: TCACGAGGAAGGAACACACGACG
Ilp1	Forward: GGTACCCAGGCATGAATCTATCCAGCG<br>Reverse: GAATTCGGAAACTGCCTCCTAGACGTT
Mop	Forward: CTCGAGATGACTGAGCTGCCATCGTT<br>Reverse: GATATCAGTTTGGATTCCGCCAGTCT
Mitf	Forward: ATGCAAGACGAGTCAGGTGTTG<br>Reverse: TCATTGGAGCTGCAGGAGATCA
Cyp26-3	Forward: AAGACTCTCTCGTCAGTCGG<br>Reverse: TGAAGGACAGCACGTCATCC
Rootletin	Forward: GAAGCGTGACACCGAGTACA<br>Reverse: TTAGCCTCGGAAAGGGCTTG

Table S2: Primers used to clone genes for riboprobe synthesis. Some primer sequences include restriction endonuclease sites.

SECTION 4: MORPHOLOGY OF *PDX* MUTANT AMPHIOXUS

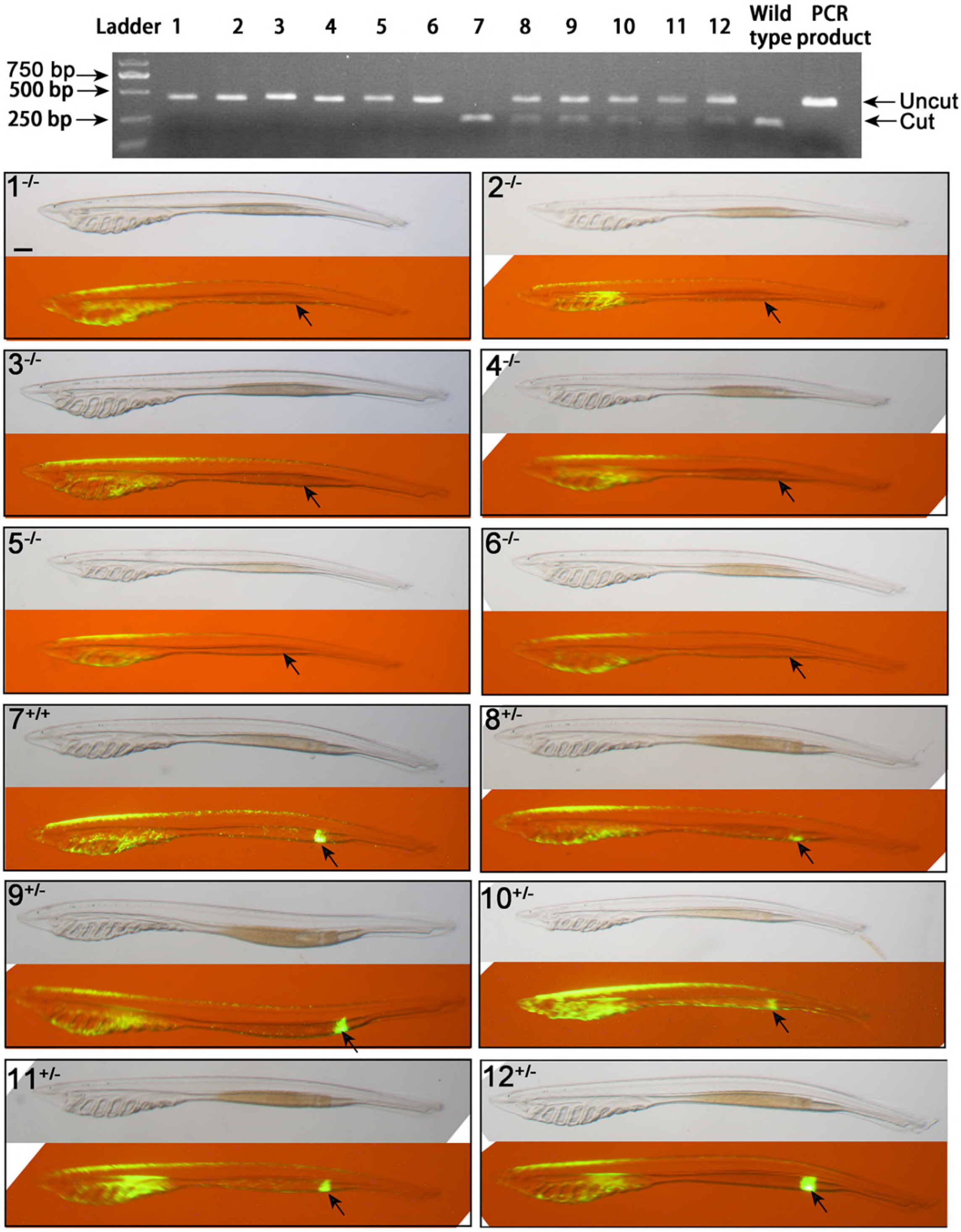

Figure S10: Amphioxus larvae at 6-7 gill slit stage under bright-field (top) and fluorescence (bottom), showing endogenous green fluorescence in buccal cirri (all genotypes) and gut region (arrow; not seen in -/- mutants). These larvae were generated by a cross between heterozygous animals with 4 bp *Pdx* deletion; larvae labelled -/- are *Pdx* 4Δ homozygotes. Anterior to the left, dorsal to the top in all images. Top panels show digestion products from PCR across the mutated region: mutation removes the restriction site, so the higher band indicates presence of the mutated allele, the lower band the wild type allele. Scale bar, 200 µm.

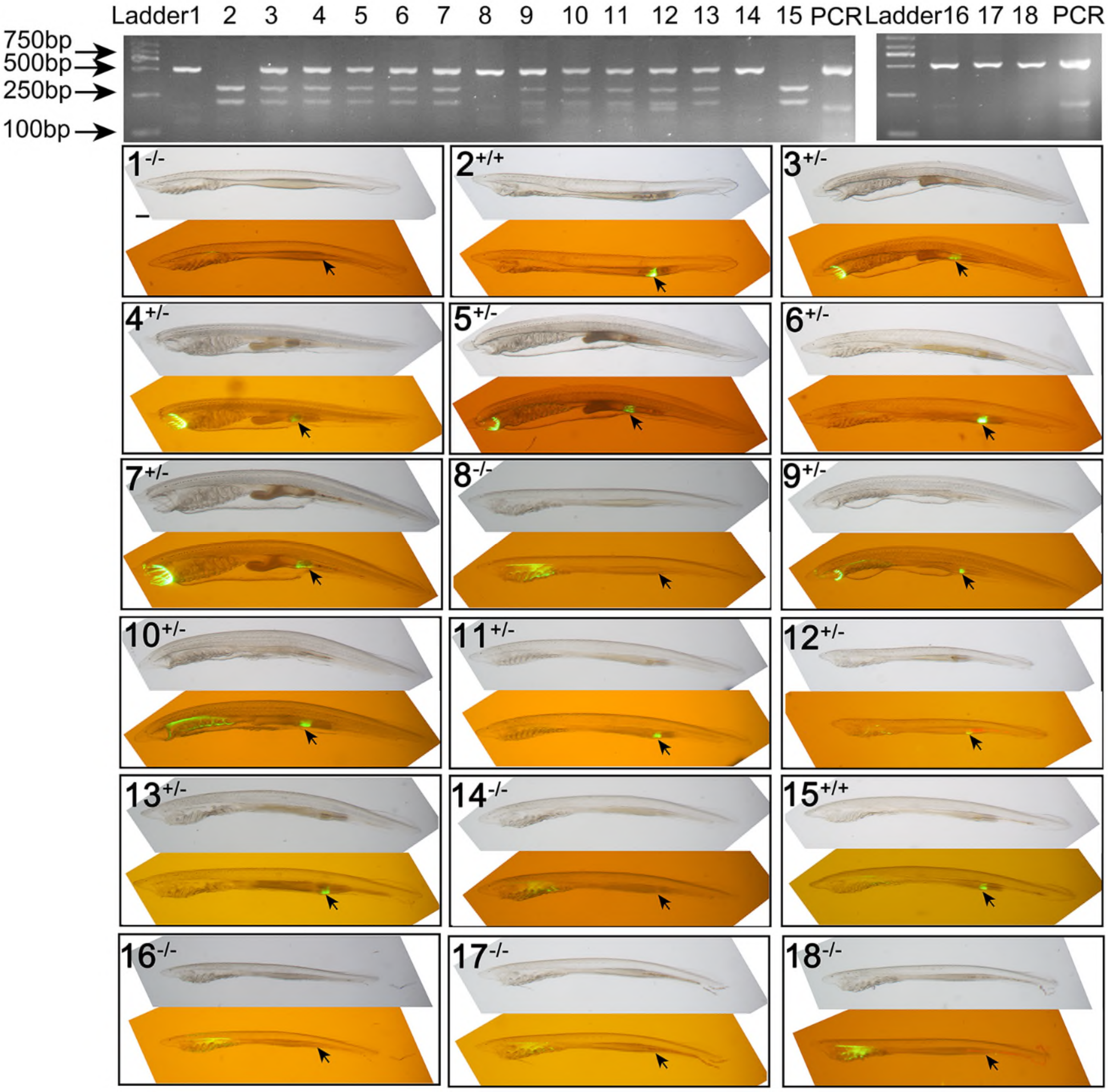

Figure S11A: Fluorescence in *Pdx* compound heterozygous amphioxus larvae and siblings; larvae imaged under bright-field (top) and fluorescence (bottom). Anterior to the left, dorsal to the top in all images. Scale bar, 200 µm.

(A) Larvae at 46 days development showing endogenous green fluorescence in buccal cirri (all genotypes) and gut region (not in -/- mutants). Larvae from a cross between female *Pdx* 11Δ heterozygote and male *Pdx* 13Δ heterozygote; the larvae labelled -/- are *Pdx* 11Δ/13Δ compound heterozygotes. Top panels show digestion products from PCR across the mutated region: mutation removes the restriction site, so the higher band indicates presence of the mutated allele, the lower band the wild type allele.

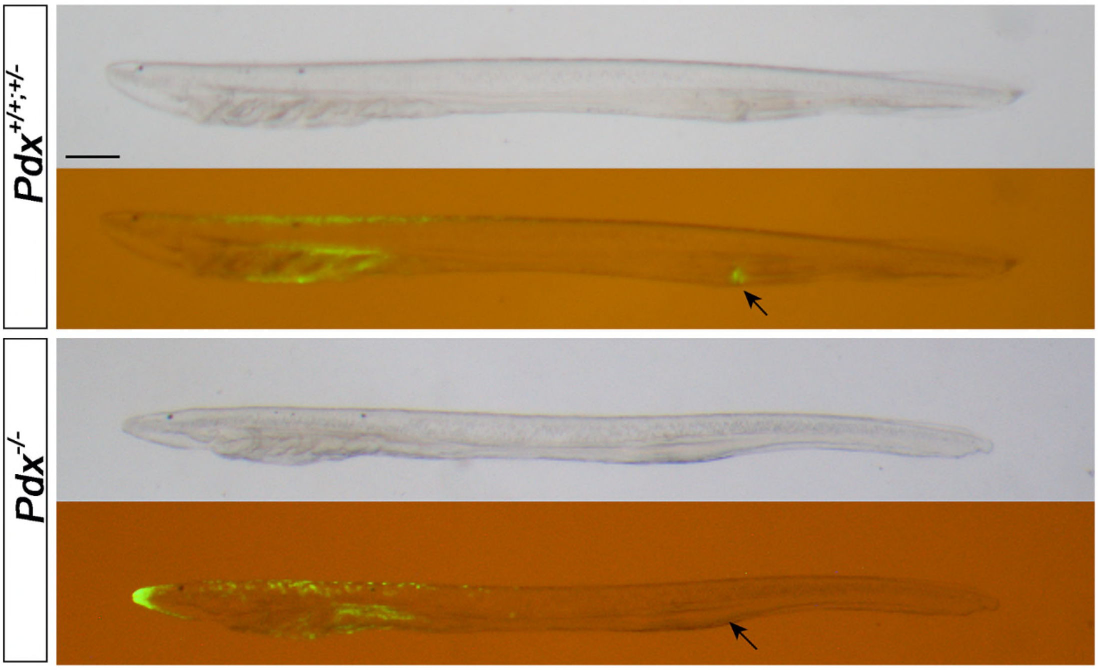

Figure S11B: Fluorescence in *Pdx* compound heterozygous amphioxus larvae and siblings; larvae imaged under bright-field (top) and fluorescence (bottom). Anterior to the left, dorsal to the top in all images. Scale bar, 200 µm.

(B) Larvae at 5 gill slit stage of development showing endogenous green fluorescence in buccal cirri (all genotypes) and gut region (arrow; not in -/- mutant). Larvae from a cross *Pdx* 4Δ heterozygote and *Pdx* 11Δ heterozygote; larva labelled -/- is *Pdx* 4Δ/11Δ compound heterozygote.

SECTION 5: MORPHOLOGY OF *CDX* MUTANT AMPHIOXUS

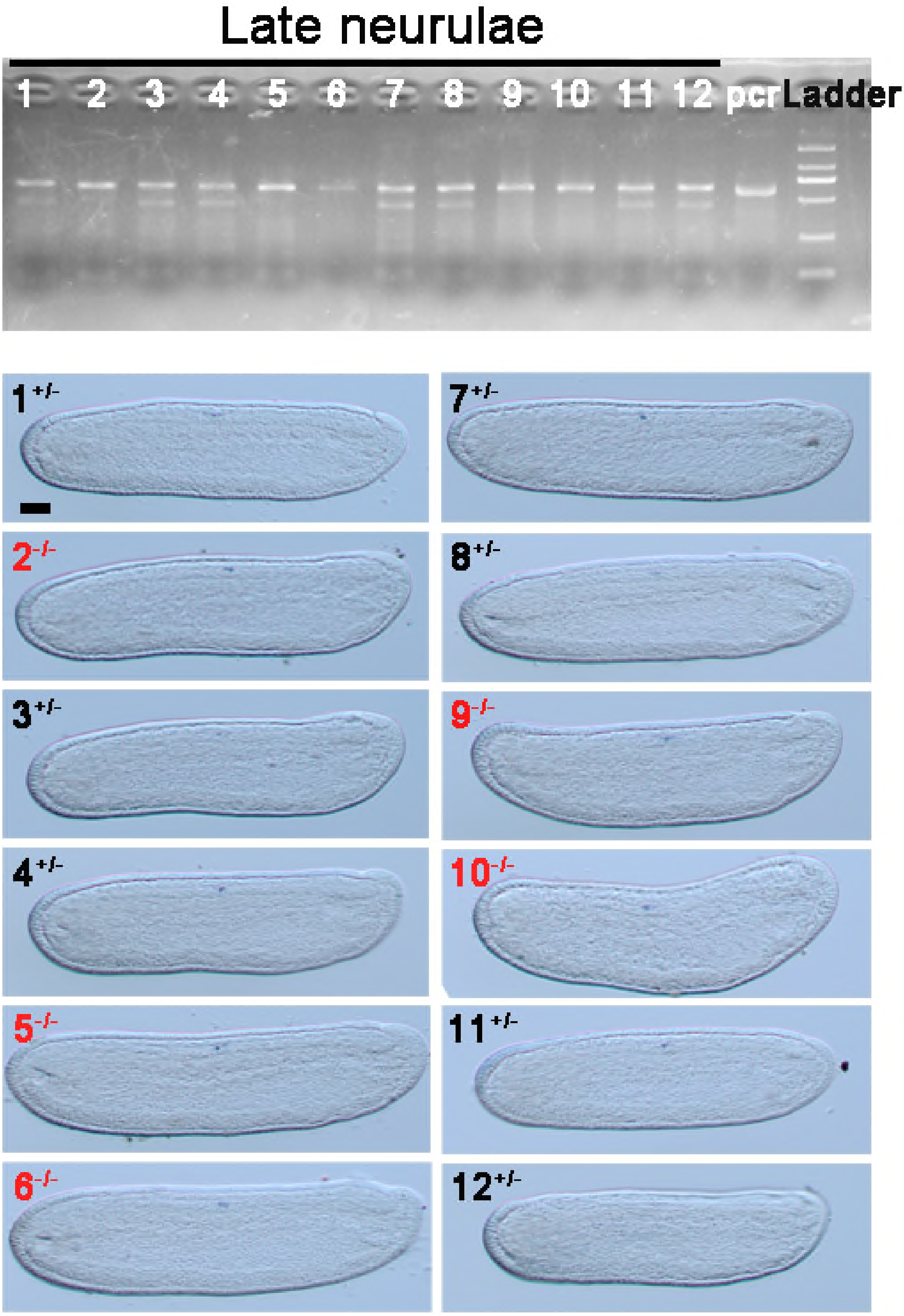

Mouth opening larvae (first batch)

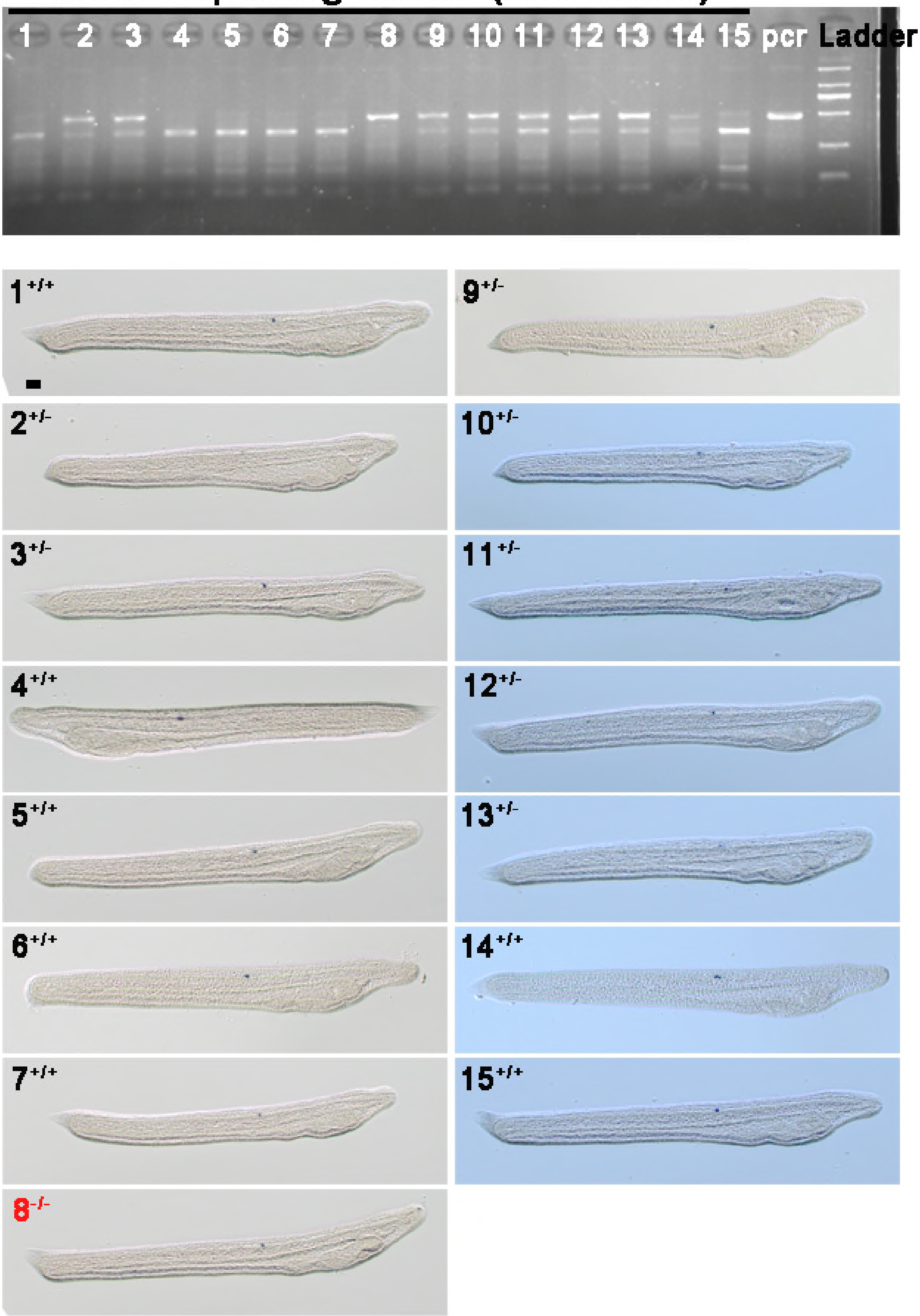

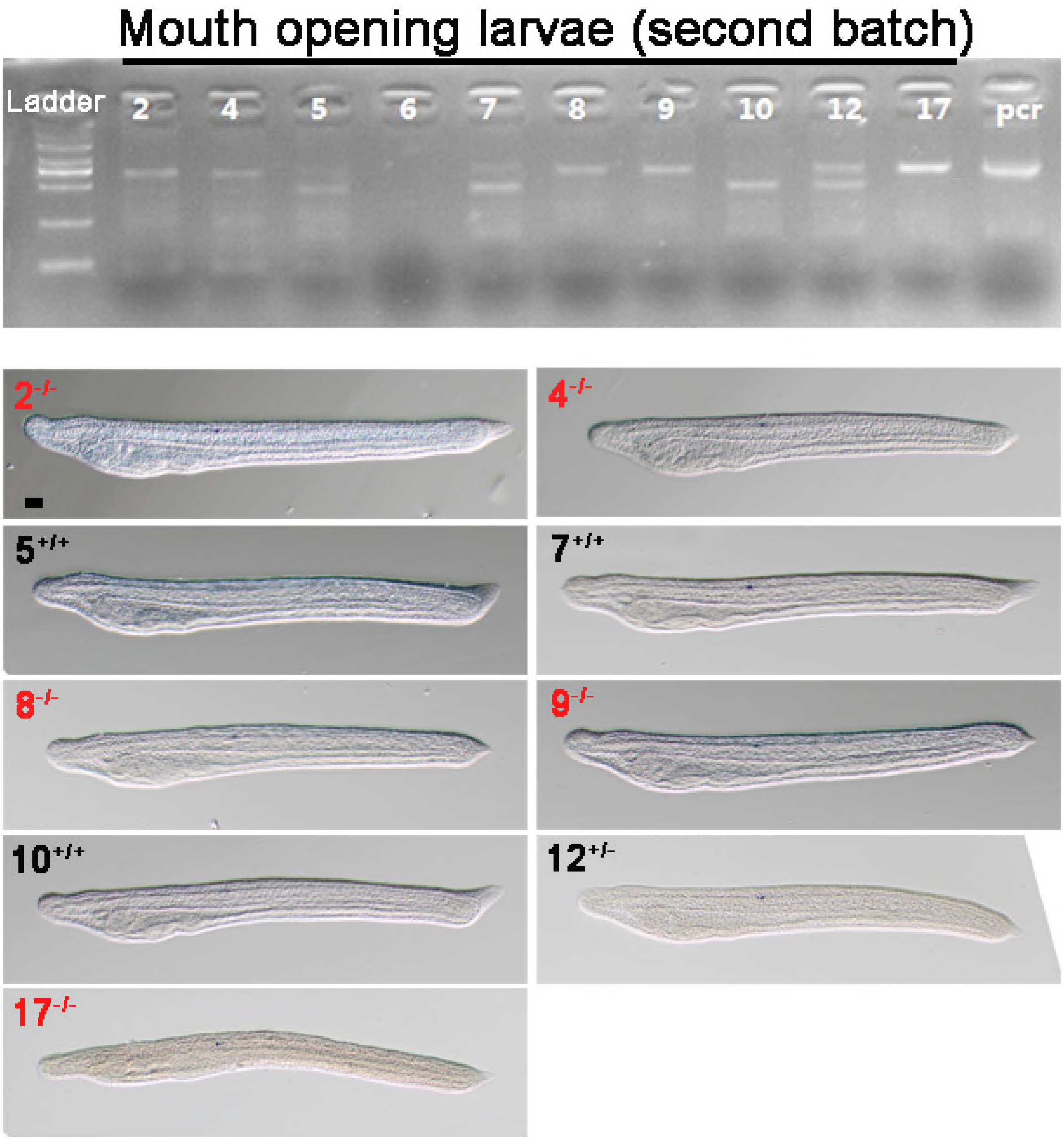

Figure S12. Morphological similarity of wild type and mutant *Cdx* embryos up to mouth opening. Top panels show digestion products from PCR across the mutated region: mutation removes the restriction site, so the higher band indicates presence of the mutated allele, the lower band the wild type allele. Late neurulae: anterior to the right, dorsal to the top; mouth-opening larvae (first batch): anterior to the right (except 4), dorsal to the top; mouth-opening larvae (second batch): anterior to the left, dorsal to the top. Scale bars, 50 µm.

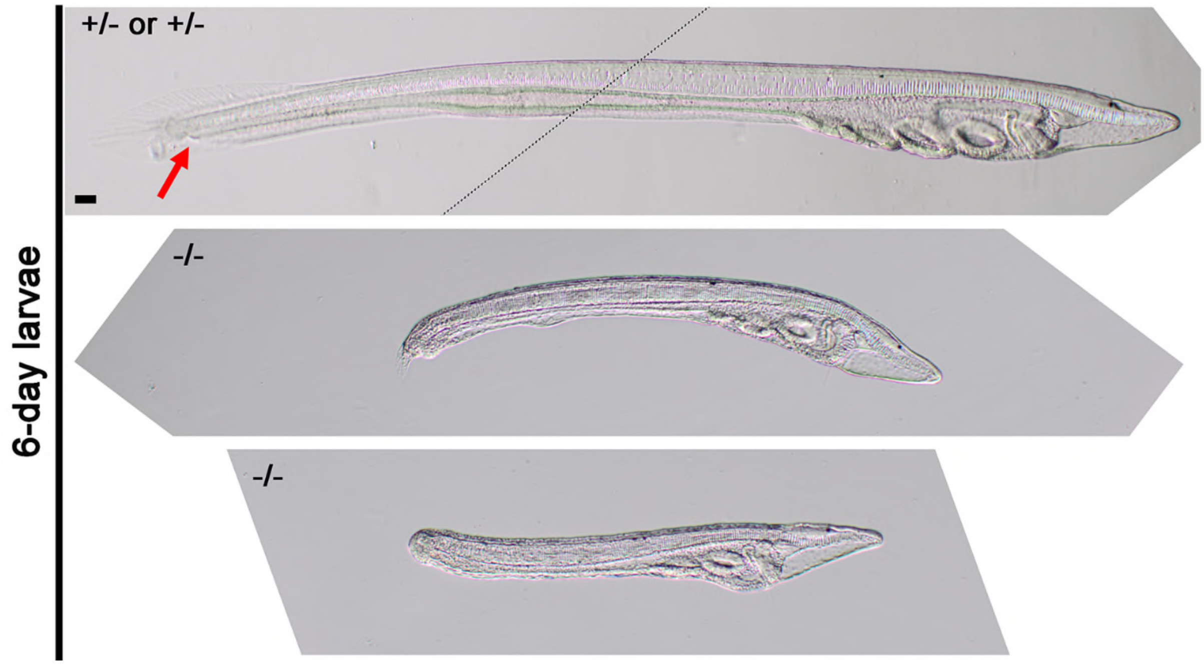

Figure S13. Extreme body truncation in *Cdx* homozygous mutants when wild type sibling larvae have reached 4 gill slit stage. Anterior to the right, dorsal to the top. Scale bar, 50 µm.

SECTION 6: ANALYSIS OF *CYP26-3*

We first tested for orthology between three *Cyp26* genes from *B. floridae* and the three reported *Cyp26* genes of *B. lanceolatum* [53].

```
>BfCyp26-1
taGGACAGAGAGAGTGGGACAGAGCAGTGCACAAGTCCGCATTTACCGACCCAGACCTTCACTTCAGACTCTCCTTGTTGCGGCCACACGATGCTGGCGGAGTTGCTGAT
TAACGCGGCAGTTCCCCTGGTGCTGGTATGGACGCTATGGACGCTGTGGAAGCACTACTCTACCCAGGGGGACCCGGCCTGTGACCTGCCCCTGCCCAAGGGGAGCATGG
GCCTGCCGTTCATAGGAGAAACCCTCGCCTTCGTCACACAGGGGGCAGACTTCAGCCGGAGCCGCCATGAGCTTTACGGAGACGTGTACAAGACCCACATCCTGGGCCGC
CCGACCGTCCGCGTCAGGGGCGCCGACAACGTGCGCAAGATTCTCCACGGCGAGAACACGCTGGTCACGACGATCTGGCCATACAGCATCCGGGCGGTCCTCGGTACACA
GAACCTCGGTATGAGCTTCGGTGAGGAGCATCGCTTTCGGAAAAGGGTCGTCATGAAAGCCTTCAACCAAAACGCTATGGAGAGCTACTTGAGATCGACTCAGACGGTCC
TGAGAGAAACAGTAGCGCAGTGGTGCGTCCAGCCGCAGCCAGTTGTCGTGTACCCGGCATCCAGAGAGATGGCGTTAAAGATCGCTGCAGCCAGCCTGATCGGCGTCCAC
ACCGGCCAGGAGGACGCCCAACGGGTCACCGTCCTCTTCCAAAACATGATAGACAATCTCTTCTCGCTACCAGTAAAGATCCCGTTCGGAGGACTTTCGAAGGCCCTGAG
GTATCGACAAATAATCGATGAGTGGTTGGAAGGCCACATCAAAAGAAAGCAGCGAGACATCGACAACGGTGACATCGGCACTGACGCCCTCTCCCGTCTGATCTTAGCGG
CGCGTGATGTCGGGCACGACCTCAACAGCCAGGAGATCCAGGACACGGCCGTGGAGCTGCTGTTTGCCGGGCACGAGACCACGTCCAGCGCCGCCACCTCCCTCATCATG
CACCTGGCGCTGCAGCCACAGGTGGTTCAGAAGGTGCAGGAGGACCTGGAGAAGCACGGGCTGCTGCAGCCGGACCAGCCTCTGAGTCTGGAGCAGGTCGGCAGGCTGAC
GTACGTGGGGCAGGTCGTCAAGGAGGTGCTCAGGATCAGTCCACCTATAGGGGGAGGCTTCAGGAAGGCTCTCAAGACATTCGAACTAGATGGATTCCAGGTTCCGGCAG
GTTGGACCGTCACGTACAGCATCCGGGACACGCACGGGTCGGTCGGTAACGTGTCCAGTCCTGACCAGTTCGACCCGGACCGCTGGGCTGCCGACAGCGATGGAAGCAGA
AGGGGGCGCCATCACTACATCCCCTTCGGTGCCGGACCTCGCGCTTGCGCCGGGAAGGAGTTCGCCAAGCTGCAGCTGAAGTTGCTGTGCGTGGAGCTGGTGAGAAGCTG
CCGCTGGGAGCTGGCGGACGGCAAGGTGCCCGCCATGACAGCTATACCTGTCCCACGGCCAGTCAATGGACTCCCTGTCCAGTTCACTCCATGCGAACCGCGAACGAACA
ACACGCTGTCAGACGCCACAGAACAGAACACAAATTTGTCAGTTTGCTACAGCAGCAACGTACCTGGTCCCAGCCACACGTCACCAAAACAACAGGACTTTGACGCTCCT
TGTCAGATTGTGATGGCACGAAAGTCGGAAGCATGCGGGGCATAGATTATGCATATAAATAAATGCCGCTGAATATAGAACTATTATATAATCATTAACCACGTGTATAT
CACGTACAACTAAAGACCTTATTTTGTTTGACAGGTCTCTATGGGAACAATTGTACATAGTATTGACCTACTCTGCGTGTGTGGACCCCTTTGTTCTCACTGTCTTATCT
CTAATAAATTGTATGATATT

>BfCyp26-2
GTAATCATTGCGACATTAACGCGGACAGCCGTCGCCATTCGGAAACAGATCAGTCGGGCGCCCATGTAGAGTTGATGGCCTACAATTTTTCACGGCGCTCGTTATGGTTT
CACCCTAGATGTTCCACTTCTAGAGAAACAGTTTCAGTGTAGGCAGAGTGAGTGAGCGGCGTCAGGGTTCAGCGCCTGGCGACAGCCATTGATCGGCTCGGGCGAGACAC
CGAGCCCGCCACGCTCTCGGCTCGTCCATCAGGCCCGCTTGGCTGCGGCAAAGGGTCACCGTCACTCCATATAAACTCCAGGCGAGCCTCTCATCGGGAGATAAAACTGC
AAAGATGCTGGTGGAGCTGGTGACTGTCCTCGTCCTGCCTTGTGTCGCTCTCCTGCTCTCCTGGAAGCTATGGACGCAGTACTACACCTGGTCCGACCCTGGCCCCGACA
CCTCCCTGCCGCCCGGCAGCATGGGTCTACCCTTCATCGGAGAGACGCTTAGCCTCGTTACGCAGGGAGGCAAATTCAGTTCCAGCAGACATGCGCAGTACGGGGATGTC
TTCAAGACCCACATCCTGGGCCGCCCGACCATCCGCGTTAGAGGGGCGACCAACGTGCGCAAGATCCTGCTGGGAGAGAACCACATCGTCACCAGTCTGTGGCCGCAGAC
GTTCCGCACGGTGCTGGGGACCGGTAACCTCGCCATGAGTAACGGCGAGGAGCACAGGCTGCGCAGAAAGGTCATCATGAAGGCCTTCAACTACGAGGCGCTGGAGAGGT
ACGTCCCCATCATGCAGGAGATCCTGCGCGAGGCTGTCCAGCGGTGGTGCGGGGCTCCCCAGCCGGTGACTGTCTGGCCCATGGCACGGGAGATGGCCTTCCGTGTGGCC
TCAGCCGTCCTGGTGGGCTTCCAGCACAGCGATGAGGAGATCCAGCACCTCACCTCTCTCTTCACCAACATGGTCAAGAACCTCTTCTCTCTCCCAGTCAAGCTACCCGG
GAGTGGGCTCAGTAACGGGCTGTTTTATCGACAAGCCATCGATGAGTGGATGATGAACCACATTCAGAGGAAGAAAGAGTTTGTGCTGCAGGGTGGCGACAGTGGAGACG
TCTTGTCGCACATCATGAACAATGCGAAGGACAACGGAGAGAAGTTGTCTGACCAGGAGATCCAGGACACGGTGGTGGAGCTGCTGTTTGCCGGGCACGAGACCACGTCC
AGCGCCGCCACCTCCCTCATCATGCACCTGGCGCTGCAGCCACAGGTGGTTCAGAAGGTGCAGGAGGACCTGGAGAAGCACGGGCTGCTGCAGCCGGACCAGCCTCTGAG
TCTGGAGCAGGTCGGCAGGCTGACGTACGTGGGGCAGGTCGTCAAGGAGGTGCTCAGGCGGCGCCCGCCCATTGGAGGAGGCTACCGCAGAGCGCTCAAGTCTTTTGACA
TCGGCGGTTTCCATGTGCCCAAGGGATGGGCGGTACTGTACAGCATCAGAGACACACACGAAGCCTCCCAAATCTTCTCCTCGCCGGAGCTGTTCGACCCTGACCGATGG
ACCCCCGAGACATCCCAGGCGCCCCTGGCCCGGTACGATATGGTGACGTTCGGCGGGGGACCACGAGCCTGTGTCGGGAAGGAGTTTGCCAAGCTCCTACTGAAGCTTCT
GTGTGTGGAGCTGACGAGAAGGTGCCGCTGGAAGCTGGCAGACGACAAGCTACCGGACATGAAGCTCATTCCCATCGTGTATCCAGCCGACGGCTTACCTGTTATCTTCA
CTCCCATTGGCGGAAAGTCACCTGGTGACGAAAACAAAAATGGCGTGCCGTATGAGGAGAGGACAAGGGGCAAGGACTGTCCTATTCTCTGCTCGGTGTCGTTTGAAAAA
GACATAAACGTCGCGACTTAAACTTTTAAGTAAAGACAAAAATATGCACATCATCTTGCCATAAGGTGTAGATAATCTTCAGAAAACGTTTATTAGTTACAGATAAGAGT
AACCAATGATTCAATTAACAATCCGGAACACAGGGTCAGACACGACAAAACTATATTTGCGATTTAAAAAAAGTATATCAATATAAAAGTAAACATTAACGCAAAGATAT
AAGAATATATACATGTGTAGAAGAAGTTAACATTTTTATTCAATACTACTTAATACTACTTAATAATTAGTATCTGAGAATGTTAAACATTAATTTGTAAGAGCAATATT
ATCCAAGGTACTGCCAGCATGCTCTCACGAGTCTGTCTCGTACATGTAAATATGCGAAAGGAACTTTTAAGCTTTTGTCATGTGTCGTGTGATGTGAATTTGGAGGAAGC
ACTAATAGCACACTCAGACTTCTGTATTACATATGGACTCCCACATTTTATTCGGAAAAGGGTACAGCTAATTTTATTTATGGTGTCGGTTACATGTTCCACAAGACGTA
GGGTTATTTGTCATAATAAGCATGTAGCCAACGGAAGACAGCAAAGTTAATGGCTAAGTATTTCGTTTGAAAATATAGGGGTGTTATTATTGACAATGTTAAAACATTGT
ATGCTAATCCAGAGAACTGAGTGCATCATGCTACATTGTGGAAATTTGGTTTAACTGCAGACTGTGTGCCTACTGGCACCGCGTAATAAATAGATACAAGACAt

>BfCyp26-3
acgactgatgacgtaatcgggcggccccgtgtcgtcgatccaaacgagctgaactctGAATGAACCACTTATTAATGACATTTGCGTGCTTGACGGGGGAATAAATAC
GAGTGAGTGGTTCTCGAGCAGCCCATTCTTGCCCAGCCTCTGTTCTACACGCAGAGGAGACTCTCCTGCTCCCGCCCGACCCCGGACCAGGCTCCCGTCATCTCACAG
CTTGTAACCACGCCACAAGACTCTCTCGTCAGTCGGCAAAATGCTGGAGGAAGTTGTAGGGTATCTTGTCTTCCCTGCCTTTCTAATGGTCCTTTCCTGGAAACTCTG
GGGGCGCTACGCCACTCCGTCGGACCCGGCCTGCGCCCTGCCTCTACCCGCGGGGACCACGGGCTTTCCGATCATCGGCGAAACTTTGTCCTTCATATTGGAGGGAGC
AGATTTCTCCAGGAAACGGCATGCCCTGTACGGAGACATCTTCAAAACCCACATCCTGGGCCGCCCGACCATCCGCGTCCGAGGCGCCGACAACGTGCGCAAGATTCT
CCGCGGAGAGAACGACATCGTGGGCACCATGTGGCCGGACAACTTCCGCATGGTGCTGGGGACGGAGAACCTGGCCATGTGTGGCAGCGGGCCGCTCCACAGGCAGAG
GAAGAAGATCGTCATGCGTGCCTTCCGCCATGATGCACTGGAGATCTACACGGACAGCATGCAGGCCATGATCGCGGACACCTTGCGGGGCTGGTGCAGGGGGCCCCA
GCCGCTGGCAGTCTACCCCGCCGCTCGAGAGATGATGTTCCGACTCGCCATAGCTGTTCTGGTCGGCTTCCATCAAGATGAGGAAGAGGCCAGGAGAGTTGGGTCACT
TTTCAGGACCGCTGTGAAGAACATCTTCTCCTTACCATTGAACGTGCCAGGAAGCGCACTCAGAAAAGCTCTGCAGTGTCGCCAGGAAATCGACGAATGGCTGAAACG
TCACATCCACGAGAAGCATGCGCAGATCTGGAGCGGAGAGGTCCCGGATGACGTGCTGTCCTTCATCATCAGCAGCGCGAAGGAGGAAGGGAAGGCTGTGGACCAACA
GCAGCTGCTGGACACGGCCGTGGAGCTGCTGTTTGCCGGGCACGAGACCACGTCCAGCGCCGCCACCTCCCTCATCATGCACCTGGCGCTGCAGCCACAGGTGGTTCA
GAAGGTGCAGGAGGACCTGGAGAAGCACGGGCTGCTGCAGCCGGACCAGCCTCTGAGTCTGGAGCAGGTCGGCAGGCTGACGTACGTGGGGCAGGTCGTCAAGGAGGT
GCTCAGGATCAGTCCACCTATAGGGGGAGGCTTCAGGAAGGCTCTCAAGACATTCGCGATTGGCGGCTTCCAGGTTCCGGAAGGTTGGGCCGTCATGTACAGCATCAG
AGACACGCACAGCGCGTCACAGCTCTTCTCGTCGCCGCAGCAGTTCGACCCGGACAGGTGGGCGGCTGCCGACAGCACGGCCATCAGGTACGACTTCTTACCCTTCGG
CGGTGGACCTCGCGCTTGCGCCGGGAAGGAGTTCGCCAAGCTGCAGCTGAAGTTGCTGTGCGTGGAGCTGGTGAGAAGCTGCCGCTGGGAGCTGGCGGACGGCAAGGT
GCCAGAGATGAAATCAGTCCCTGTTCTTCATCCCGCCAATGGACTTCCGGTAAACTTTGTCTCTTTGGATGACGTCACTGTCAAACGTGAGGATGCAGACGGACTTGC
TGCTCCCGCGCATGCTCCGTTGATGAACACCGACCTGGTCACGAGAAGTGACCCCTGTTTGACCTTGGACAAAAACGGGAACTTGTATCCGACCAGCGAGCAAAACTC
CCCGGACACTGTGACGGTGGTTGGACCGGACTTGTCCAACATTGTCTAAATGGAACTGGAAAAAAAGCCAGTCGATTGTAGTAATTAGGAAGCTAGCTCTCTATCAAC
TTATGCATATTGACAAACACACCCACACATGTACACACGCAACCACCCATATGCACACACACACACATGCACGCACGCACCCACCTACATGTACACGCACAAATGC
GCATACCTTCACACACACACGCACACACACACACGCACACACACACACGCACACACACACACACACACGCACACACACACACACACACACACACACGCACCAAC
ACCCAGAGTTGTGTGTAATCAGAAAACTTTGAAGCGAAACATGAACCTGTTGAAAGGACGCATGGCTTGTGGTCAACTTGCCAAACCTTGTAAATATGACCTTATGTC
AATGCTGTTACGTTATCAGGTTTGATTACTTCATCTAAATGCAAGCATCTATGGTACATGTACAACTTGTGAATATGGGATTGTGCTTGTGGCCTAGCAATGATGACA
ATCACTACCCCCACTAAGACCAATGCTTGTTTAAAACTTGATGTATGTCATTAAAAGGTGGGACTTAATTAAATACCCAGTATATAGTTGCATGCTGATATTTATTTA
TCATATACAGCACAAAACTATTTATTTTATTACAACACCACTTATTTATATGGATCATTTTTATACGTTTAGTTGTGGAGCACTGTCTATTTTTATGACAATCATACT
ATGACAACCATGCGTTATATGGCTATATAATGTTATAATAAAGTTATCAATCT
```

Figure S14: DNA sequences of the deduced transcribed region of three *Cyp26* genes from *B. floridae* (Bf). Blue text = untranslated regions (UTR), red text = coding sequence, underlined sequence = region used as in situ hybridisation probe for *Cyp26-3*.

We constructed a phylogenetic tree, which confirmed clear orthology between *Cyp26-3* genes of *B. floridae* and *B. lanceolatum* with 100% support.

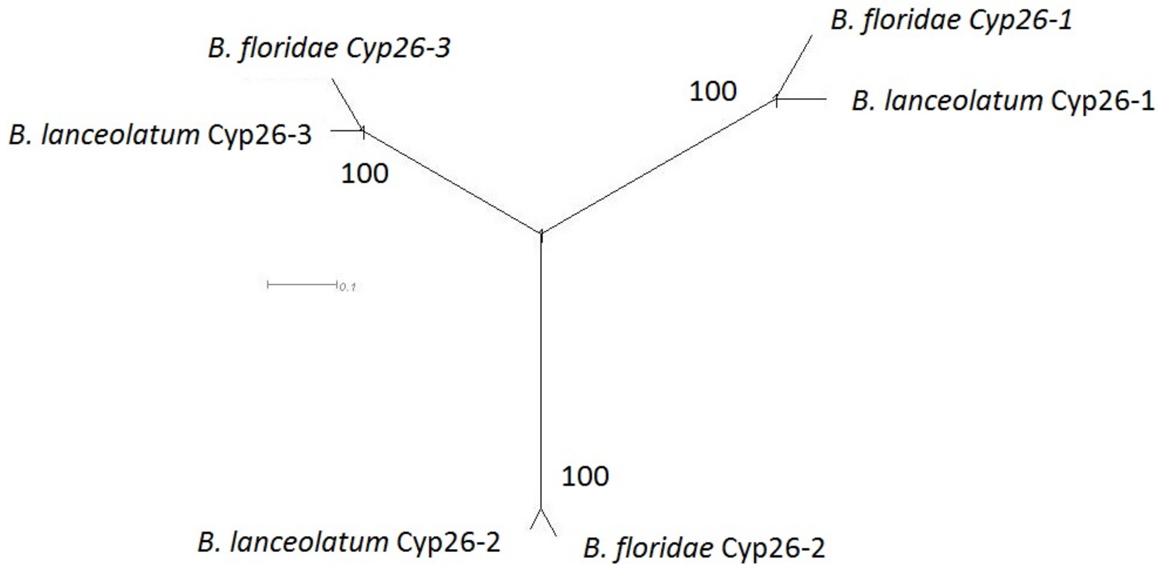

Figure S15: Unrooted phylogenetic tree demonstrating orthology between *Cyp26* genes of *B. floridae* and *B. lanceolatum*. Deduced amino acid sequences were aligned using Clustal Omega 1.2.4 and a Maximum Likelihood tree built using PhyML (PhyML 3.1, WAG matrix, 100 bootstraps).

We identify five putative Cdx binding sites (TTTATT/AATAAA) in ~3.1 kb upstream of the ATG of *Cyp26a-3* (blue highlights in Figure S16). Four are upstream of the deduced transcriptional start site (black text) and one in the 5' untranslated region (blue text). Two were chosen for mutagenesis (bold and underlined); these have a purine residue following TTTATT and best match mouse Cdx binding sites (NTTTATDRBHB; [58]).

```
BfCyp26a-3 promoter

TGGTGAGATTCGTGCGTACCGTCAAAGGACTTCTGTGATATCTATTTCTAACAAGGATACATCGCTCTTGTTCAGAAATAGGATCTTGGCAA
TGGCAATAAGTACCAAAAGTATATATTATACGTTATGCAATACATTACAGTTGATACGACTAAGACGTATTTCCAAGATTTTGACGACAGTG
GGTGGTAGACTTTAGGAAGACACTGGTGTCTATAGTCTCCAGGTAGACGTGTCAGATGGATGAAAACAGAACAGTCTGGCCAAATAGGGACC
AATAAATTGTCATGGGATTTCCAGTAAGTCTTTTGCAGTCTGTTGAGCTATCTATACGCCGGCCGGTCAGTTAACTCCTACTCCTAGGCCAG
TTGATAATAACTCTTACTACCACTTAACTAGGCTTTTATTTCAAGCTTATCCAAAGTCCCCTGCCCTGTCACTATTCAGTcagtagtctggt
agagattcTAACatatttaattttttgttgtgtctCACATGGTGTTTTAACATACACTGTTTCAGCATACTCAATCAAACCACGACCTGACG
TCCAGTCACTTCTTATGATACATTACAAGCTAtatgccaccaaaaaatcaagaccatagcatgtccaggtcaaaagatacaaaaagtcgttc
tgctgcagtaccaaggtcacaaaccagggtgcccaaaatcaccctgaccttcgtctttgcaatacctacccacatatcaaatatcattataa
ccCAACCAgagttatggtgaccacaaatatccggaaacacaaacagacacacgcatagacacacacacagaaacaccaaaaactatgcctcc
atttttcatggaggtaacaatctGAAGAGACCTCAAAGCTGCAGACGGCAAGTGGCCATGTTACTATGATTGTTAGGTGCAGCTCAAACAGC
CCAGAtgttgacctgtgacctttacTAATTAGCTGTGAGACCGTTGTAGAAAGGTGGCTGCAACCGTGTTCCAAGAAGTTCTACGGATATTG
AGTCAGAGGAAAAAGACTTTGTACAAAGTGTTGAAGAACTTCTTCTATTTTATCATGACATATTCTGCTGAGCAGTGGGTAGTCGACATCCC
AGTTGTCATAGCAAGAGGACAGGCtcagatcccccccccccccccatacgaTACTGAAGCTGTTCTATATCATACATATCATGCAATTATG
TCTATTGATGATCTAGAAAGCTCAAAGTCTAGCACGACAAAAGCGGACAGAGTTTGTTAACAACCTTTCCGTCTCACTAACTTCGCTGTTCT
CCGCACAACTTTAACGTTATCTGTTTCGAAGAAGGCATAGGATTCATTTTTAGAGTGAACAAAGATATCGACGACGTTTTCCCCTCAAACTT
GTACCTATAAAAGAGAACGTACAGACAGGGGTTACTTCCGGTTAAGGGccctctttttgataagtgctgTAAAGTGGCGTGGCACTTGAGGA
GGGTTATCAGAAATTGTCAGTAGGCTCGAGGTCAATAACCCCCACCCACTTCACACGAGGACTCTCTCAGGAGATGTCTATTCCGATCTCTG
CAAATAGAATAGACAGGAGAACATTGCTCAGCGGTTACGGAACCGCAGTTTCTGCTTGATGTCTATGTATGATCCGCGGCGCTGAGTGCGTT
TCAGCTGCACTATTAAACGCCACTGCAATgatgttcgccttgcattcggttgGTGGATCCCTGGCCAAGCCATGCGCTATGTCAAAGAGACT
TTAGATATGGTACtttctgctttctctgcttagagaaagcagcatgtgagCAAGAATTTGAGAAGGAgcatggtagttgaacacacaccact
accagtggactagccccctgctgtaatgattacaaaagttgtgtggcccggggctacagaaacggagatgggtgccgtcctatgcaccatct
ggtacGGGAAGGACTTCAACTTACCTTAAGTAATGCCGTGTGGCTGATTAGGGTATCTGGTAACTTTCGaaaaaaaGAGAGTAAAGAGAGGA
AAATTATTTTCCCTCATAAAAACCCAATTAGCATTCCAGCGGAAGGTTACACGGCAAGCCATATTTATACAAAGAACGCCAATCATGTTAAT
ACCAGTATTACATTGATTATTTCATCTTCTCTGAAGACCTTTGATATACCAATAGTACAATAAGCCGTAAAGTTCGAACGTTCGCAACACCA
GGTACCCCTAGCAGAAAGAGATGAACTTTTGCAGTGTATCAAACATTGTGTTTTCATTGCCAATGACTTTCacgatatatttttttcaagac
gTTAGGTATAcccacgggggggggggggggttctgttGTGGTCCACAGAGAGACAGACGTCGGAGTTAAAAACTACGCGCACTACTGTCTAC
TGACCCCGACAGAAACCTTCAGTATACCCCGACCTACGTGGTGGGGTAGCGGAGTGATGACAGAGACCCGGGTTTGACCCCTTGCACTCAGC
AACACATCCAATGTTTATTTGCTTCTCTCGTTATTCTGGGAATCTAGCAAAGATGACTAGTCGTGACAGCGCCCTGGCAGATAATTGACAGG
CCACTACAATTAAAGTCCGTGTGTGAGCGATGGGCCGGGAGGTCAGGCAGGGCGTTGGCTTGTCTCTTGTCTGTCAGTCGGGAGGTTTGCAC
TCAGGAGCCAGCAAATAACGTAACATGTCCGTTCACTCCACCTGTCAATATTCATTAGTCCCTCGGCGCGGGCATTGCACTTATTTTATTAG
CCGCGGTCCAATTGGACCTCTGTCAGCCAATCGGCGTGGTCATTACAGGCGGTTCTTCAGACGACAAGGGGCGTGGCCTTGTGGGAGGTACG
ACTGATGACGTAATCGGGCGGCCCCGTGTCGTCGATCCAAACGAGCTGAACTCTGAATGAACCACTTATTAATGACATTTGCGTGCTTGACG
GGGGAATAAATACGAGTGAGTGGTTCTCGAGCAGCCCATTCTTGCCCAGCCTCTGTTCTACACGCAGAGGAGACTCTCCTGCTCCCGCCCAA
CCCCGGACCAGGCTCCCGTCGTATCACAGCTTGTAACCACGCCACAAGACTCTCTCGTCGGTCGGCAAAATG
```

Figure S16. DNA sequence upstream of start codon of *B. floridae Cyp26a-3* gene. Red ATG = start codon; blue text = 5' untranslated region; blue highlight = putative Cdx binding site; underlined blue highlight = binding sites chosen for mutagenesis; yellow highlight = cloning primers.

The 3.1 kb region was cloned into pGL3 basic vector (Promega) between *Sac*I and *Nhe*I sites, and the resultant construct ('Cyp26-3 promoter') was used to make mutant constructs with one or two putative Cdx binding sites mutated ('BS1 mutation', 'BS2 mutation' and 'BS1,2 mutation') using a PCR method (Table S3). Controls were empty pGL3 vector and not injected samples.

Genes	Primer sequences (5'→3')
Cloning primers	Forward: TGGTGAGATTCGTGCGTACC<br>Reverse: CTCTCTCGTCGGTCGGCAAA
Binding site 1 mutagenesis primers	Forward: TCTGGCCAAATAGGGACCgAcgAcTTGTCATGGGATTTCCAG<br>Reverse: CTGGAAATCCCATGACAAgTcgTcGGTCCCTATTTGGCCAGA
Binding site 2 mutagenesis primers	Forward: CGCGGGCATTGCACTTATcTgcTgAGCCGCGGTCCAATTGGA<br>Reverse: TCCAATTGGACCGCGGCTcAgcAgATAAGTGCAATGCCCGCG

Table S3: Primers used for cloning and mutagenesis of *Cyp26-3* promoter sequence

Injection solutions were prepared containing 3 ng/µL Renilla luciferase vector pRL-TK (Promega), 20% glycerol, 5 mg/ml Texas Red dextran, with or without 30ng/µL each of above luciferase constructs. Microinjection into unfertilized amphioxus eggs was conducted as previously described [87]. For each experiment, ~60 embryos were collected at 16 hours post fertilization. Wild type embryos from the same batch were also collected and used as a negative control ('WT'). Levels of luciferase and Renilla were detected with the Dual Luciferase Kit (Promega Co.) using a GloMax luminometer with an integration of 10 seconds. The level of luciferase activity was normalized to the level of Renilla activity for each experiment. All experiments were repeated three times (Table S4).

**Construct**	***Cyp26-3* promoter**	**BS1 mutation**	**BS2 mutation**	**BS1,2 mutation**	**pGL3**	**WT**
RFV1	16.0989	8.0092	8.3821	4.8000	0.5515	0.4338
RFV2	20.2335	4.8345	6.5707	3.1083	0.4145	0.3469
RFV3	11.6292	7.4662	5.4355	4.8504	0.4836	0.3582

Table S4: Raw values of relative luciferase for each construct and three replicate experiments RFV1 to RFV3. Values compared graphically in Figure 5B.

SECTION 7: DIFFERENTIAL EXPRESSION OF CANDIDATE TARGET GENES

Raw Illumina sequence data are deposited under NCBI BioProject PRJNA594548 [96], including 12 BioSamples (SAMN13521182 to SAMN13521193) and 12 SRA datasets (SRR106745850 to SRR10674591). Expression data inferred by read mapping, and DNA sequences of superTranscripts referred to below, are given in Additional file 2: Supplementary Data (Tabs 2 to 7).

(a) Principal Component Analysis

We used Principal Component Analysis (PCA) to test for outliers and batch effects in the RNAseq replicates before testing for differential gene expression between mutant and control sets. PCA used the plotPCA function of the DESeq2 v3.8 R package [95]. This uses the counts obtained from the featureCounts [94] function of the Subread R package and stores it in the dds object; plotPCA uses data from the rlog transformation of dds.

PCA applied to transcriptomes from the *Cdx* experiment separated samples according to batch or developmental age along Principal Component 1 (Figure S17 A). Specifically, batch 1 samples (collected at 34 h post-fertilization) were separated from batch 2 samples (collected at 42 h post-fertilization) along PC1. In contrast, Principal Component 2 separated samples according to genotype (mutant vs. wild type); Figure S17 A.

Distance matrix analysis also separated batch 1 and batch 2 samples (Figure S17 B).

We therefore conducted two Differential Gene Expression analyses, focussing on Batch 2 only (42 h embryos; results in Additional file 2, the results below, and results discussed in main text) or combining all samples (34h plus 42h embryos; results in Additional file 2, plus noted in *Cyp26-3* analysis below).

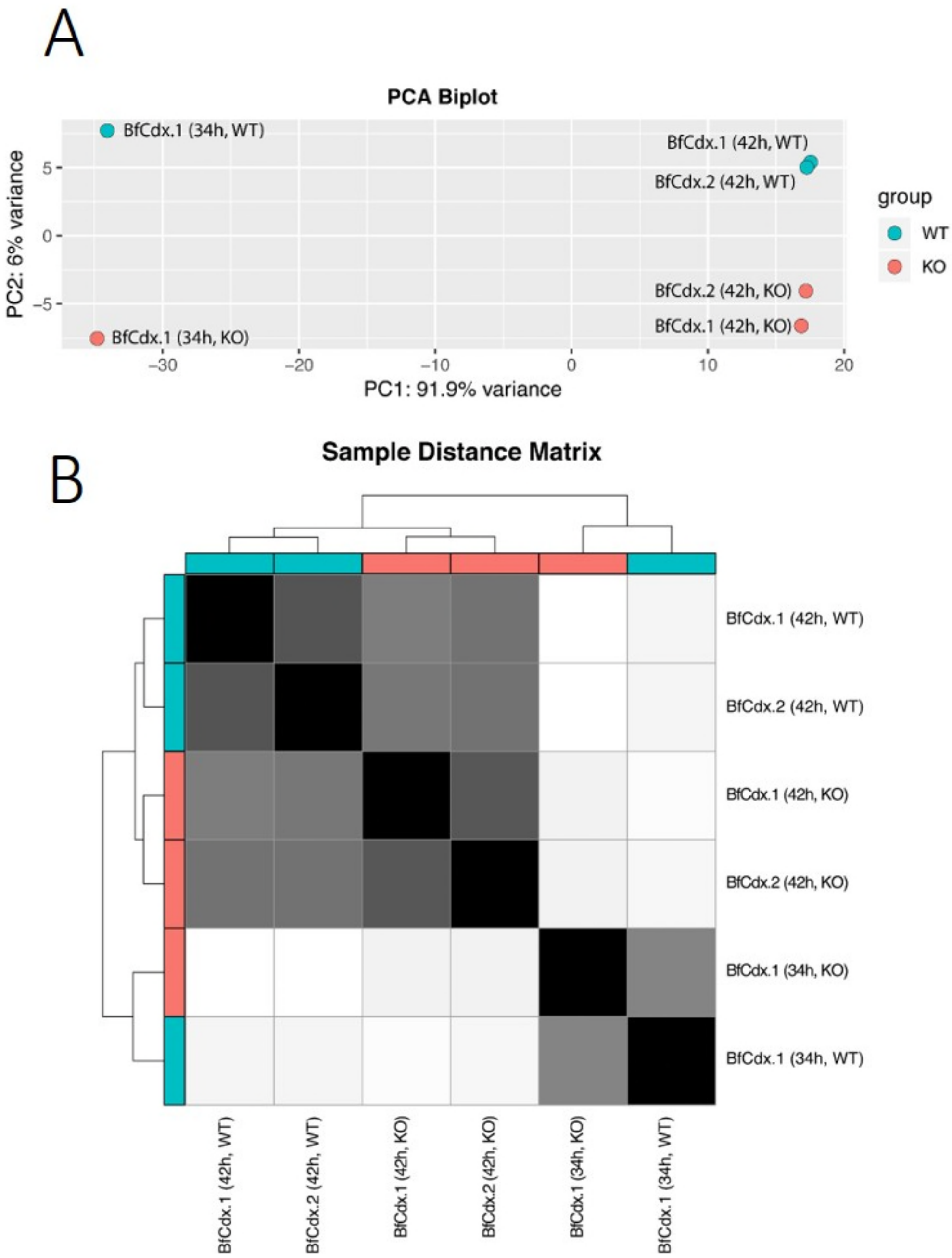

Figure S17: Principal Component Analysis (PCA) and Distance Matrix analyses of *Cdx* mutant (KO) and control wild type (WT) RNAseq data sets. (A) PCA Biplot of *Cdx* mutant and wild type RNAseq data sets. The samples collected at 34 h post-fertilization (points towards left side of PCA Biplot) are separated along Principal Component 1 from those collected at 42 h post-fertilization (points towards right side of PCA Biplot). PC2 separates mutant and wild type samples. (B) Distance matrix analysis and dendrogram comparing *Cdx* mutant and wild type RNAseq data sets.

PCA applied to transcriptomes from the *Pdx* experiment separated samples according to genotype along Principal Component 1 (mutant vs. wild type). One wild type sample (BfPdx.2 WT) grouped aberrantly, especially along Principal Component 2, and was excluded from Differential Gene Expression analysis as a putative outlier (Figure S18).

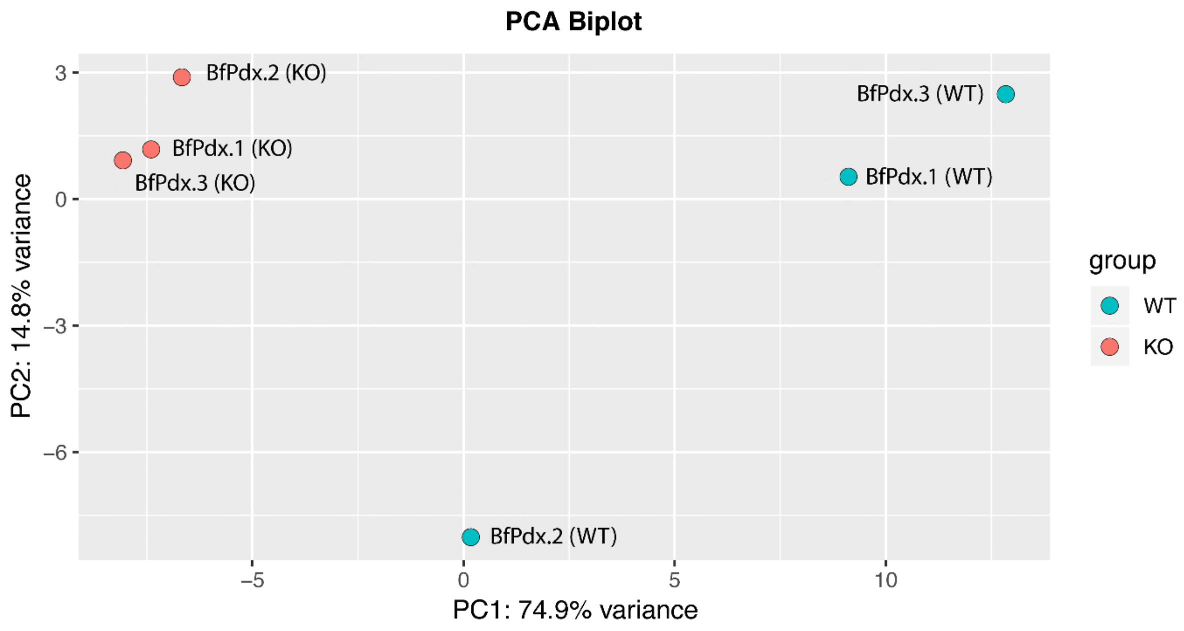

Figure S18: Principal Component Analysis (PCA) Biplot of *Pdx* mutant and wild type RNAseq data sets. Principal Component 1 primarily separates samples according to genotype (wild type vs. mutant);wild type sample 2 (BfPdx.2 WT) is a putative outlier.

.

(b) Analysis of GFP genes affected by mutation of amphioxus *Pdx*

In *Pdx* 4Δ/11Δ mutants, we found down-regulation of reads mapping to 11 contigs from the GFP gene family (GE_G14886, FE_G16436, NOVEL_103923, NOVEL_102722, NOVEL_50813, ML_G28518, FE_G16645, FE_G16414, ML_G19054, ML_G18969, NOVEL_74108). To analyse whether these are variants of the same amphioxus gene, or multiple genes, NCBI blast align (nucleotide vs nucleotide) was used to search a database of DNA sequences of the open reading frames of the 13 amphioxus GFP open reading frames described by Li et al. [63].

Contig	Best hit	Weaker hits	Log 2 fold change	adjp
>FE_G16436 (short)	GFP-8	GFP-11	-1.59	3.05E-10
>NOVEL_103923	GFP-13	GFP-10,8,12	-2.08	4.00E-20
>NOVEL_102722	GFP-13	GFP-10,8,12	-2.21	1.21E-20
>GE_G14886 (short)	GFP-1	GFP-2	-0.64	0.009
>NOVEL_50813	GFP-10	GFP-8, 13, 12	-0.68	0.002
>ML_G28518	GFP-10	GFP-8,13,12	-0.68	0.004
>FE_G16645	GFP-8	GFP-10,13,12	-0.95	7.23E-07
>FE_G16414	GFP-10	GFP-12,9,1	-1.06	0.04
>ML_G19054	GFP-10	GFP-8,13,12	-1.26	2.59E-09
>ML_G18969	GFP-13	GFP-8,10,12	-2.05	5.78E-05
>NOVEL_74108	GFP-13	GFP-10,8,12	-3.06	1.30E-14

Table S5: GFP contigs affected in *Pdx* mutant transcriptomes, showing results of blast align, compared to expression fold change data from DEG analysis.

The most likely identities for these contigs are *GFP-8, GFP-10* and/or *GFP-13*. The single match to *GFP-1* is weaker and derived from a short contig so is less reliable. It should not be concluded that all three best hit genes (*GFP-8, GFP-10* and *GFP-13*) are changing in expression level, however, because four amphioxus genes - *GFP-8, GFP-10, GFP-12* and *GFP-13* - have highly similar nucleotide sequences and are the product of recent tandem gene duplication [63]. Short read sequence data, as generated in this study, cannot be unambiguously assigned to a particular gene and reads will be split by multimapping between them. We conclude that one, or more, of the genes *GFP-8, GFP-10, GFP-12* and *GFP-13* has been down-regulated in expression following *Pdx* mutation.

(c) Analysis of insulin signalling pathway genes affected by mutation of amphioxus *Pdx*

i. Signalling peptides. We found three contigs with partial sequence similarity to insulin-like peptide (*ILP*) genes (FE_G15481, GE_G16157 and FE_G15511); these are expressed ~2 to 11 fpkm. NCBI blastn analysis vs nr database (31/7/19) suggests the first two represent genes related to *ILP*; the third (FE_G15511) is true *ILP*. None of these contigs showed significant expression level changes in *Pdx* mutants (Additional file 2).

ii. Binding proteins. SuperTranscript M_G27744 encompassed three related transcripts (Table S6), each with stretches of 100% identity to a gene annotated as insulin-like growth factor binding protein 7 (*IGFBP7*) in *B. belcheri* (XM_019790737, LOC109486832) and its

homologue in *B. floridae* (XM_002608818.1). These contigs showed clear down-regulation in *Pdx* mutants. A second superTranscript FE_G33063 also matched this gene and showed similar down-regulation (Table S6).

Contig	Transcript	Best hit	Log 2 fold change	adjp
ML_G27744	MLTU49440	IGFBP7	-0.67	0.002
	MLTU49442	IGFBP7	-0.73	0.0008
	MLTU49437	IGFBP7	-0.70	0.004
FE_G33063		IGFBP7	-0.62	0.0015

Table S6: IGFBP contigs affected in *Pdx* mutant transcriptomes

However, there are additional contigs with sequences matches to IGFBP genes (for example, FE_G30801, GE_G21098, ML_G26668) or IGFBP acid labile subunits (GE_G18919, ML_G15423) that do not show differential regulation

iii. Receptors. Contig FE_G26674 encoding insulin-like peptide receptor (100% blastx hit to *B. floridae* ILP receptor BRAFLDRAFT_128184 XP_002585764.1) showed up-regulation in *Pdx* mutants (log 2 fold change 0.49, adjp = 0.009). The *B. lanceolatum* orthologue of this gene (second blastx hit), has been shown to bind peptides of insulin and an ILP analogue when expressed in cell culture, supporting its designation as an insulin-like peptide receptor [64].

(d) Analysis of iLBP, RAR, RXR, Cyp26, Rootletin and T-box gene expression in amphioxus *Cdx* mutants

In *Cdx* $^{-/-}$ mutants (42h analysis), we found 1.6- to 13.5-fold down-regulation of reads mapping to eight contigs from the intracellular lipid-binding protein (iLBP) gene superfamily which in vertebrates include CRABP (Cellular retinoic acid-binding protein), CRBP (cellular retinol-binding proteins) and FABP (Fatty acid-binding proteins). These contigs are FE_G16051, GE_G22703, ML_G19061, NOVEL_10269, NOVEL_50856, NOVEL_65559, NOVEL_74162, NOVEL_82533. To analyse whether these are variants of the same amphioxus gene, or multiple genes, BLASTX was used vs NCBI nr (30-31/7/2019). This revealed that seven contigs represent the gene *iLBP4* and one represents *iLBP6*.

The first seven contigs match *iLBP-4* of Albalat et al. [56], NCBI XP_002607338. The same gene was named *CRABP* by Jackman et al. [66], NCBI AAQ72814.1, but does not group more closely with vertebrate *CRABP* in phylogenetic trees when a diversity of amphioxus *iLBP* genes is included (see Supplementary Figure S6 of [56]. The last contig matches *iLBP-6* of Albalat et al. [56], NCBI XP_002607336.

In *Cdx* $^{-/-}$ mutants, we found no significant change in expression level of *RAR* (contig FE_G19398), *RXR* (contigs FE_G39642, ML_G28820), putative *RALDH* genes (multiple contigs), Wnt genes (multiple contigs) or *Cyp26-2* (contig FE_G28942); all values in Additional file 2: Supplementary Data (Tab 3). *Cyp26-3* is not present in the main 42 h transcriptome dataset analysed due to low expression; in the mixed 34 h/42 h dataset it shows up-regulation (contig FE_G28864; adjp 0.0005), albeit from a very low initial expression level (1.3 mean fpkm to 2.4 mean fpkm; Additional file 2: Supplementary Data, Tab 4). This may seem contrary to the in situ hybridisation findings; however, the transcriptomes are

from a later developmental stage and so this effect likely reflects the feedback loop between increased RA signalling and *Cyp26* gene expression first described in zebrafish [59-61] and confirmed here in amphioxus (see main text).

Rootletin gene expression is clearly down-regulated in the transcriptome of *Cdx* $^{-/-}$mutants, indicating that once down-regulated in mutant embryos it does not recover at later developmental stages. We found 7 contigs for the same gene, matching different isoforms or incomplete transcripts (ML_G1957, ML_G8904, ML_G8919, NOVEL_38192, GE_G1583, GE_G1727, NOVEL_60824). All were significantly down-regulated in *Cdx* $^{-/-}$mutants. The longest contigs are summarized in Table S7.

Contigs for most T-box genes showed no significant change in expression level, apart from *Brachyury-2* (1.46 fold up-regulation, from 6.8 mean fpkm in wild type to 9.9 mean fpkm in *Cdx* mutants; Table S7).

**Contig**	**Best hit**	**Log 2 fold change**	**adjp**
FE_G16051	iLBP-4	-2.20	1.54E-22
GE_G22703	iLBP-4	-2.06	4.20E-15
ML_G19061	iLBP-4	-2.40	2.92E-09
NOVEL_50856	iLBP-4	-2.11	5.05E-14
NOVEL_65559	iLBP-4	-2.42	9.08E-61
NOVEL_74162	iLBP-4	-2.67	1.06E-14
NOVEL_82533	iLBP-4	-3.77	0.015
NOVEL_10269	iLBP-6	-0.70	0.002
ML_G1957	Rootletin	-0.6	~2E-08
ML_G8904	Rootletin	-0.6	~1E-05
FE_G44204	Brachyury-2	0.545	0.004

Table S7: Intracellular lipid-binding protein, Rootletin and T-box contigs affected in *Cdx* mutant transcriptomes

(e) Hox gene expression in wild type and *Cdx* mutants

In previous work from our laboratory and that of H.V. Isaacs (University of York, UK), we showed that disruption on *Cdx* function in *Xenopus tropicalis* has a colinear-like effect on Hox gene expression [19]. In addition to the expected down-regulation of posterior Hox genes after *Cdx* disruption, we detected higher expression of anterior Hox genes (consistent with *Cdx* genes activating posterior Hox genes and repressing anterior Hox genes in normal development). We wished to test if an analogous colinear-like relationship to *Cdx* occurred in amphioxus.

First, we note that absolute levels of expression differ greatly between Hox genes in wild-type embryos, as estimated from transcriptome read mapping (calculated here from the *Cdx* experiment 42h samples). Only *Hox1*, *Hox3*, *Hox4* and *Hox6* have expression levels above 10 fpkm; *Hox2*, *Hox5* and *Hox7* have fewer reads counts and the more ‘posterior’ paralogy group genes barely any (Table S8; Figure S19).

Hox gene	Contig	Mean fpkm wild type	Mean fpkm *Cdx* mutant	adjp	Log2FoldChange
Hox1	HOX1	16.51668	23.66045	0.000546	0.518436
Hox2	HOX2	1.85504	1.780453	0.999998	-0.05927
Hox3	HOX3	22.38144	21.47403	0.999998	-0.05965
Hox4	AB028208.1	28.09742	23.44197	0.192511	-0.26125
Hox5	HOX5	1.842797	0.953663	0.672645	-0.9493
Hox6	HOX6	24.91482	14.74147	6.16E-05	-0.75688
Hox7	FE_G13616	0.241352	0.063064	0.073839	-1.93772
Hox9	FE_G13248	0.059181	0.057971	NA	-0.02971
Hox15	FE_G13062	0.043841	0.152279	0.266805	1.795219

Table S8: Hox gene expression changes in *Cdx* mutant transcriptomes

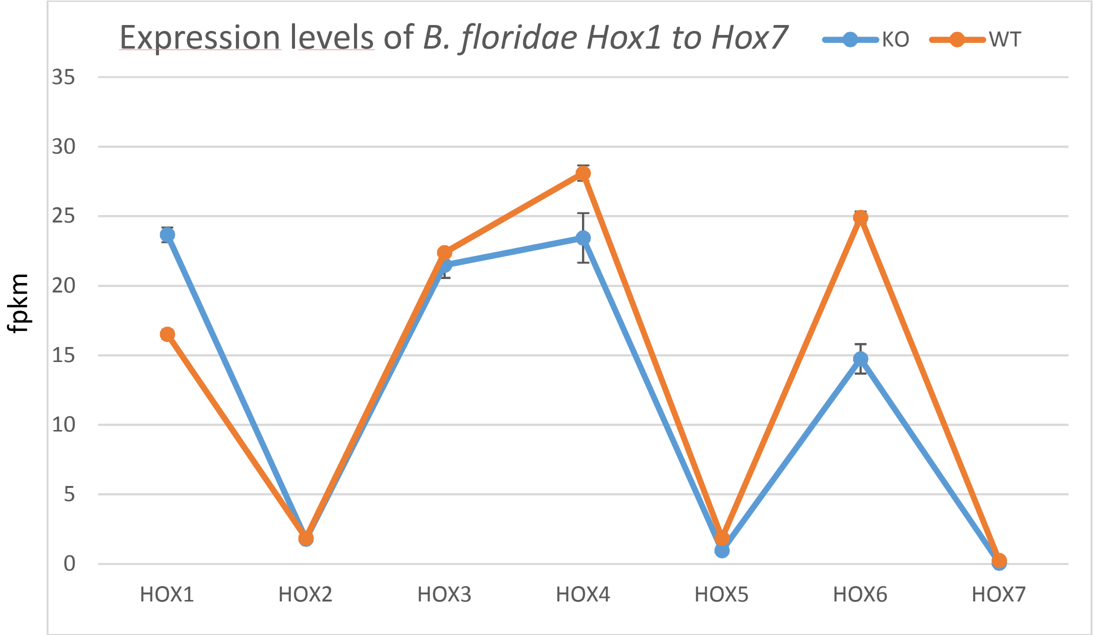

Figure S19: Line graph showing expression levels (FPKM) of Hox genes in *B. floridae* determined from 42h RNAseq data generated in the present study. Blue line, homozygous mutant; orange line, wild type and heterozygotes. Mean values +/- standard deviation shown.

This finding is consistent with in situ hybridisation experiments by Wada et al. [70] to wild type *B. floridae* embryos, which detected strong signals for *Hox1*, *Hox3* and *Hox4*, but very weak expression of *Hox2*, although contrary to the stronger *Hox2* pattern reported by Schubert et al. [71]. The read counts reported here for *B. floridae* are also consistent qualitatively with *B. lanceolatum* transcriptome data (from a slightly later developmental stage) reported by Marlétaz et al. [69], extracted and plotted below, apart from higher expression of *Hox1* in *B. lanceolatum* (Figure S20).

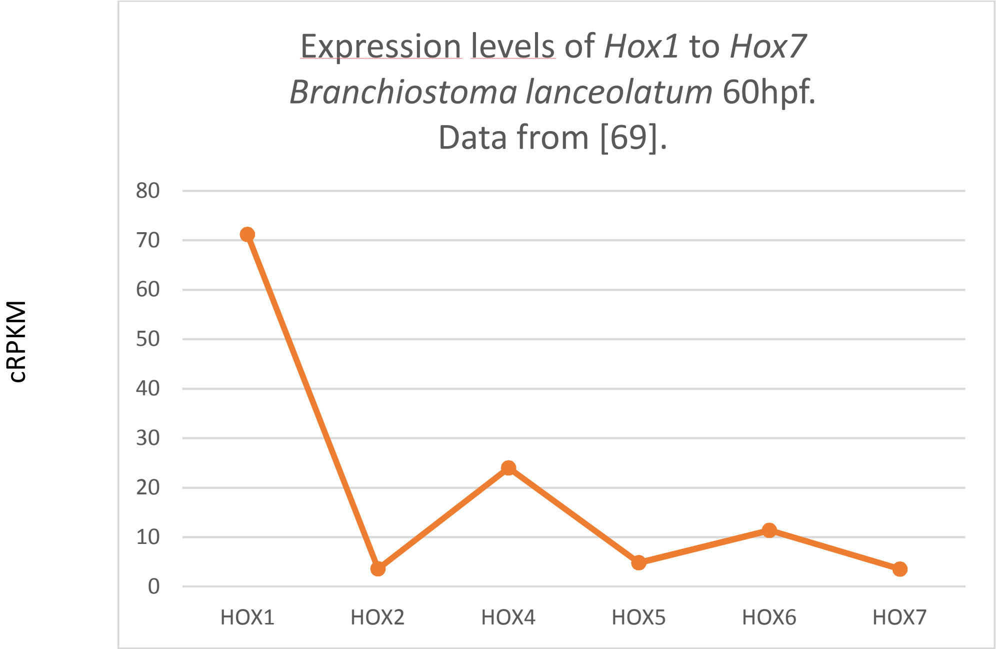

Figure S20: Line graph showing expression levels (cRPKM) of Hox genes in *B. lanceolatum* determined from RNAseq data of Marletaz et al. [69]

Second, we detect a colinear-like response of Hox genes to mutation of *Cdx*, with paralogy group 1 gene expression higher in mutants, *Hox2*, *Hox3* and *Hox4* unaffected, *Hox5* and *Hox6* mildly down-regulated, and *Hox7* strongly down-regulated (Table S8). Only the *Hox1* and *Hox6* expression changes are significant when each gene is considered one at a time (Table S8); considering genes as a cluster and plotting mean changes collectively reveals a significant negative slope to the response (Figure S21).

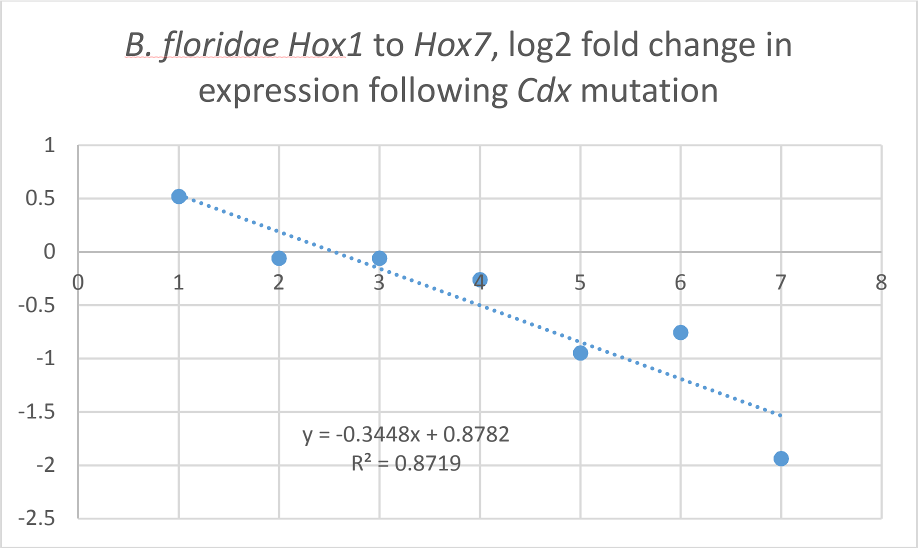

Figure S21: Expression level change (fold change log 2 scale) in Hox gene expression in Cdx homozygous mutants compared to wild type and heterozygotes.

SECTION 8: DIFFERENTIAL EXPRESSION OF GUT-ENRICHED GENES

Gut-enriched genes were identified from published *B. lanceolatum* RNA-seq data (NCBI GEO GSE106430; [69]). We defined a gene as gut-enriched if it had a higher mean expression level in gut than in any other adult tissue (eggs and embryos excluded) and if expression level in gut was at least twice the expression level in seven out of eight other tissues (neural tube, muscle, gill bars, hepatic diverticulum, testis, ovary, skin, cirri; Additional file 2: Supplementary Data, Tab 8). This gave 2083 *B. lanceolatum* gut-enriched genes (listed in Additional file 2: Supplementary Data with expression levels; Tab 8 and Tab 9). These were matched to contigs in the current study using blastn with an e-value cut off of 1^{e-70} giving 4705 gut-enriched *B. floridae* superTranscripts, also listed in Additional file 2: Supplementary Data, Tab 9.

We asked if the genes affected by *Pdx* or *Cdx* mutation were enriched in gut-enriched genes. There are 5831 superTranscripts that are expressed differentially between wild type and *Pdx* mutant larvae; of these 482 are also found in the set of 4705 gut-enriched contigs. Similarly, there are 1428 superTranscripts expressed differentially between wild type and *Cdx* mutant larvae; of these 218 are found in the set of 4705 gut-enriched contigs. This equates to 8.3% of the *Pdx* differentially expressed contigs and 15.3% of the *Cdx* differentially expressed contigs.

To test if 8.3% and 15.3% represents enrichment, we ran 1000 simulations of each sampling (5831 or 1428 sequences chosen randomly from the superTranscriptome) and assessed overlap with the gut-enriched dataset. Mean overlaps were 3.3%, with the experimental data being a highly significant outlier in each case (arrows in Figure S22). Hence, mutation of *Pdx* or *Cdx* has a disproportionate and significant effect on expression of gut-enriched genes.

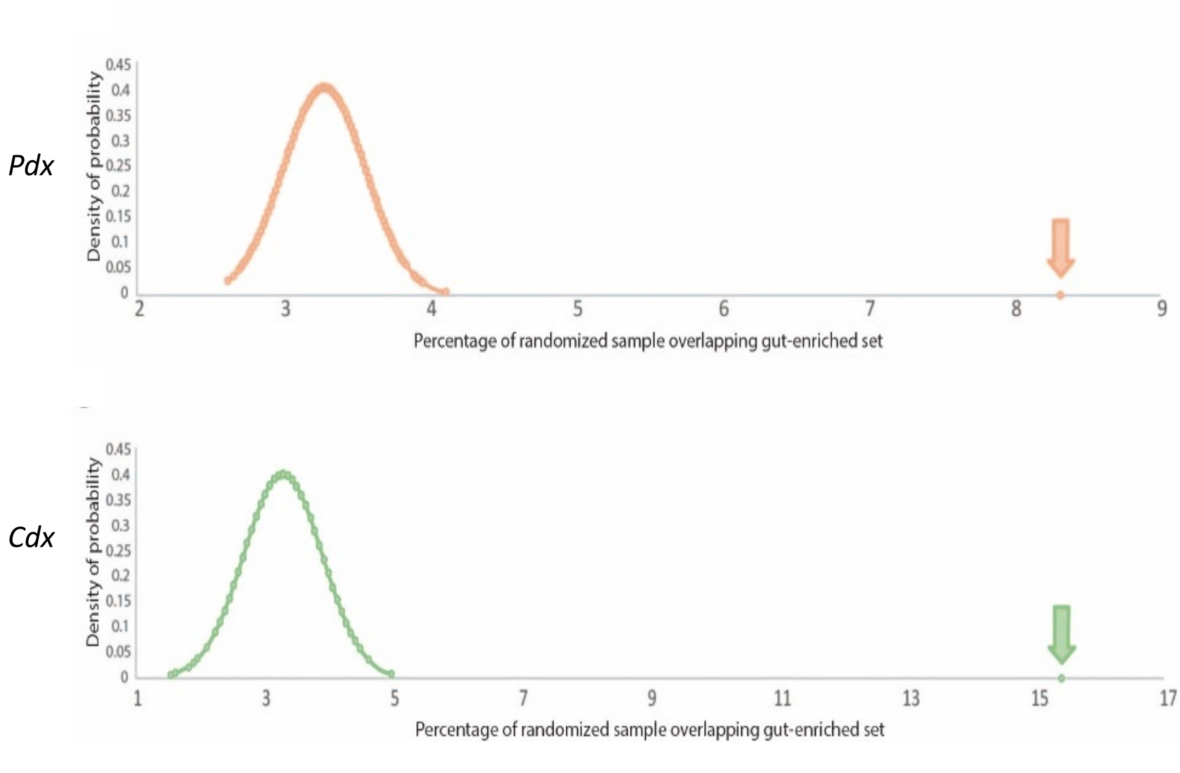

Figure S22: Mutation of amphioxus *Pdx* or *Cdx* has a disproportionate effect on gut-enriched genes. The degree of overlap (orange and green arrows) is outside the range of overlap generated by random sampling (orange and green curves).

We also searched the 'gut-enriched, differentially expressed' data sets for genes implicated in gut cell function. To do this, we first reduced the data sets to well-annotated protein-coding genes by (a) clustering highly similar sequences (>90%) by cd-hit-est, and (b) excluding genes without protein-coding annotations in the IncDNA-BF database [92,93]. This reduced the *Pdx* 'gut-enriched, differentially expressed' from 482 contigs to 218 genes, and the *Cdx* 'gut-enriched, differentially expressed' from 218 contigs to 92 genes. Putative identities are given in Tab 10 and Tab 11 of Additional file 2: Supplementary Data. Examples from the *Pdx* data set are given in Table S9.

Putative gene name	Putative function	Contig	Mean fpkm wild type	Mean fpkm Pdx mutant	adjp	Log2FoldChange
**Chymotrypsin**	Digestive enzyme	ML_G10589	108	71	0.005	-0.607
		FE_G7789	80	52	0.018	-0.607
**Mucin**	Coats mucosal epithelia	GE_G2301	4.7	2.5	0.004	-0.912
**Proton-coupled folate receptor**	Intestinal absorption of dietary folates	FE_G867	3.0	4.4	0.011	0.574
**Brush-border sucrase-isomaltase/maltase-glucoamylase (SI/MGA)**	Intestinal digestion of dietary starch	NOVEL_52564	2.7	4.1	0.021	0.615

Table S9: Selected gut-associated gene expression changes in *Pdx* mutant transcriptomes

Citation numbers refer to references cited in main manuscript
